# Supplementary material for: Exploring the In Vivo Existence Forms (23 Original Constituents and 147 Metabolites) of Astragali Radix Total Flavonoids and Their Distributions in Rats Using HPLC-DAD-ESI-IT-TOF-MSn
Source: Molecules. 2020 Nov 26;25(23):5560. doi: 10.3390/molecules25235560 (PMC7729672; doi:10.3390/molecules25235560)
Supplement: Supplementary file 1 [file molecules-25-05560-s001.pdf]

molecules

Supplementary Materials

Exploring the In Vivo Existence Forms (23 Original Constituents and 147 Metabolites) of Astragali Radix Total Flavonoids and Their Distributions in Rats Using HPLC-DAD-ESI-IT-TOF-MS^n

Li-Jia Liu, Hong-Fu Li, Feng Xu *, Hong-Yan Wang, Yi-Fan Zhang, Guang-Xue Liu, Ming-Ying Shang, Xuan Wang and Shao-Qing Cai *

State Key Laboratory of Natural and Biomimetic Drugs, School of Pharmaceutical Sciences, Peking University, No. 38 Xueyuan Road, Beijing 100191, China; lijialiu@bjmu.edu.cn (L.-J.L.); 1716383012@bjmu.edu.cn (H.-F.L.); wanghy@pku.org.cn (H.-Y.W.); zhangyf0911@pku.org.cn (Y.-F.Z); guangxl@bjmu.edu.cn (G.-X.L.); myshang@bjmu.edu.cn (M.-Y. S); xuanwang6818@bjmu.edu.cn (X.W.)

* Correspondence: xufeng76@bjmu.edu.cn (F.X.); sqcai@bjmu.edu.cn (S.-Q.C.); Tel.: +86-10-8280-2534 (F.X.); +86-10-8280-1693 (S.-Q.C.)

1. Supplementary Methods

1.1. Detailed Information on the Determination of the Contents of ARTF and its Major Constituents

1.1.1. ARTF Content Determination by HPLC-DAD-ELSD

ARTF content determination was performed on a Shimadzu Prominence LC-20A liquid chromatograph system coupled with a low temperature ELSD, consisting of a DGU-20A_3 degasser, an LC-20AD binary pump, an SIL-20A autosampler, a CBM-20A communications bus module, an SPD-M20A diode array detector, a CTO-20A column oven, and a low temperature ELSD-LT II detector. The chromatography separations were performed on an Industries Epic C18 column (250mm × 4.6 mm, 5 μm) (New Brunswick, NJ, USA) protected with an Agilent ZORBAX SB C18 guard column (12.5 mm × 4.6 mm, 5 μm) (Santa Clara, CA, USA). The mobile phase consisted of 0.1% formic acid (A) and acetonitrile (B) at a flow rate of 1.0000 mL/min. A gradient elution program was adopted, specifically as 10–20% B at 0–10 min, 20–30% B at 10–55 min, 30–40% B at 55–65 min, 40–60% B at 65–105 min, 60–100% B at 105–115 min. The injection volume was 10 μL and the concentration of ARTF was 10 mg/mL. The column temperature was maintained at 35 °C, and the DAD detection wavelength was 190–400 nm. The temperature for ELSD was set at 40 °C; the gas pressure was 348 Kpa, and the gain value was set at 10. The peaks appearing both in UV at 254 or 280 nm and ELSD were regarded as flavonoids, and peaks only appearing in ELSD were considered as non-flavonoids.

The content of ARTF and six main constituents were calculated using area normalization of ELSD chromatogram.

1.1.2. Analyzing the Constituents of ARTF

ARTF was analyzed by HPLC-DAD-ESI-IT-TOF-MS^n, and the conditions were the same as described in Section 3.5 of this paper. The major constituents were identified by comparison with reference compounds. Some of the low content constituents were identified by the interpretation of their LC-MS^n data.

1.2. Detailed Isolation Procedure of Compounds from Urine

ARTF-containing urine extract obtained in Section 3.3 (ca. 750 g), was dissolved in 1.5 L deionized water, filtered and then subjected to XAD-2 macroporous resins column chromatography.

Water, 20% methanol-water, 60% methanol-water, and 100% methanol were used to elute the column in sequence, and each elution volume is 4 column volumes (4BV), and concentrated to dryness to get Fraction 1 to Fraction 4, and their weight were 658.3 g, 6.9 g, 15.8 g and 2.5 g, respectively. Compound analysis of these four fractions was performed on HPLC system, and the conditions were the same as described in Section 3.5 of this paper. By comparing the chromatogram of each fraction with that of blank urine (Figure S31–S34), we could know that Fraction 1 and Fraction 2 were mainly endogenous ingredients of urine, and ARTF constituents and metabolites existed in Fraction 3 and Fraction 4. Fraction 3 was dissolved in 10% methanol and filtered, then subjected to an ODS column. 10% methanol-water to 100% methanol were used to elute the column gradually to get 408 fractions, and the similar fractions were combined to get 16 parts. Part 6 was 1.7 g and it was dissolved in 40% methanol-water, filtered and separated by an ODS column, and the resulting Fr.12–14 was separated by Sephadex LH-20 to obtain 2.67 mg MI-1 (M108). The precipitation of Part 7 was 0.89 g and it was separated by a Shimadzu preparative HPLC system to get 818.30 mg MI-2 (M32) and 16.66 mg MI-3. Part 7 was 1.9 g and it was dissolved in 40% methanol-water, then filtered and separated by an ODS column; the resulting Fr.11 was separated by a Sephadex LH-20 column and a Shimadzu preparative HPLC for several times, and 34.62 mg MI-4 (M106) was obtained. At the same time, other 8 compounds were also isolated, but their structures could not be elucidated. The isolation procedure was shown in Figure S35.

2. Supplementary Results

2.1. LC-MS^n Data of Detected New Compounds

Table S1. LC-MS^n data of detected new compounds.

NO.	t_R (min)	Formula (M)	Ion	Identification	Ion Fragment
**M62** △,★	26.975	$C_{22}H_{24}O_{14}S$	$[M-H]^-$	Tetrahydrocalycosin glucuronide sulfate	[543.0792]: 463.1248(2.80), 368.0474(6.03), 367.0470(100), 287.0836(14.38); 272.0544(2.80)
**M63** △,★	32.35	$C_{28}H_{32}O_{17}$	$[M-H]^-$	Tetrahydrocalycosin diglucuronide	[639.1607]: 464.1227(5.84), 463.1243(100), 287.0914(7.62)
**M69** △,★	27.675	$C_{16}H_{16}O_{12}S_2$	$[M-H]^-$	Hydroxy tetrahydrocalycosin disulfate	[463.0017]: 383.0453(100); [383.0453]: 368.0208(44.011), 303.0880(100), 288.0649(100), 275.0934(23.10), 151.0417(13.82), 137.0308(13.82)
**M70** △,★	45.733	$C_{18}H_{20}O_{10}S$	$[M-H]^-$	Dihydroxy dihydrocalycosin sulfate	[427.0687]: 412.0488(71.89), 347.1084(10.48), 332.0910(100), 317.0683(3.05), 302.0426(1.74), 285.0388(1.74), 275.0204(12.67), 137.0641(3.47); [332.0910]: 317.0699(100), 285.0308(59.81), 137.0287(29.99)
**M104** △,★	38.25	$C_{23}H_{26}O_{14}S$	$[M-H]^-$	Astraisoflavan glucuronide sulfate	[557.0997]: 477.1395(17.38), 382.0695(10.99), 381.0658(100), 302.1112(1.9), 301.1067(40.2), 286.0856(2.74), 254.9833(1.27)
**M105** △,★	52.51	$C_{23}H_{26}O_{14}S$	$[M-H]^-$	Astraisoflavan glucuronide sulfate 1	[557.0997]: 477.1485(5.06), 382.0643(7.01), 381.0630(100), 301.106733.22)
**M129** △★	49.09	$C_{20}H_{22}O_{12}S$	$[M-H]^-$	Tetrahydrogenistein pentose sulfate 2	[485.0787]: 406.1226(5.93), 405.1187(34.28), 274.0812(8.9), 273.0771(100),
**M130** △,★	39.85	$C_{21}H_{24}O_{13}S$	$[M-H]^-$	Tetrahydrogenistin sulfate	[515.0887]: 435.1319(100), 273.0792(94.34), 241.0022(30.78)
**M136** △,★	32.293	$C_{21}H_{22}O_{12}S$	$[M-H]^-$	Equol glucuronide sulfate 1	[497.0786]: 417.1194(20.36), 321.0411(100), 241.0898(3.94); [321.0411]: 241.0882(100), 121.0407(5.30)
**M137** △,★	28.292	$C_{21}H_{22}O_{12}S$	$[M-H]^-$	Equol glucuronide sulfate 2	[497.0806]: 417.1231(1.82), 321.0404(100); [321.0404]: 241.0945(20.99), 135.0488(13.48), 121.0323(100)
**M142** △,★	26.3	$C_{27}H_{30}O_{17}$	$[M-H]^-$	Tetrahydro trihydroxyisoflavone diglucuronide 1	[625.1440]: 450.1091(10.57), 449.1094(100), 273.0777(19.40)

**M143** △,★	26.867	$C_{27}H_{30}O_{17}$	$[M-H]^-$	Tetrahydro trihydroxyisoflavone diglucuronide 2	[625.1413]: 450.1107(7.51), 449.1073(100), 273.0738(23.21)

△ New metabolites found *in vivo* after administration of ARTF; ★ Potential New compound by retrieving information from SciFinder database.

2.2. Tables for the Distribution of In Vivo Original Constituents or Metabolites of ARTF, for the Constituents of ARTF, and for Pharmacological Effect of In Vivo Compounds of ARTF

2.2.1. Distribution of Original Constituents

Table S2. The distribution of original constituents after administration of ARTF in 10 organs of rat.

No.	t_R (min)	Formula (M)	Ion	Identification	Heart	Liver	Spleen	Lungs	Kidneys	Stomach	Intestine	Colon	Thymus
**F1** ♥	58.827	$C_{16}H_{12}O_5$	$[M-H]^-$	Calycosin	▲	▲	▲	▲	▲	▲	▲	▲	▲
**F3** ※	56.485	$C_{16}H_{12}O_5$	$[M-H]^-$	Calycosin isomer 2						▲			
**F4** ♥	27.200	$C_{22}H_{22}O_{10}$	$[M+HCOO]^-$	Calycosin-7-O-glucoside						▲			
**F6** ♥	71.750	$C_{16}H_{12}O_4$	$[M-H]^-$	Formononetin	▲	▲	▲	▲	▲	▲	▲	▲	▲
**F7** ※	66.802	$C_{16}H_{12}O_6$	$[M-H]^-$	Pratensein/Rhamnocitrin /5,7,4'-trihydroxy-3'-methoxyisoflavone						▲		▲	
**F8** ※	68.197	$C_{16}H_{12}O_6$	$[M-H]^-$	Pratensein/Rhamnocitrin /5,7,4'-trihydroxy-3'-methoxyisoflavone						▲		▲	▲
**F11**	54.377	$C_{15}H_{10}O_4$	$[M-H]^-$	Daidzein	▲	▲	▲		▲	▲	▲	▲	▲
**F12**	55.485	$C_{15}H_{12}O_4$	$[M-H]^-$	Trihydroxychalcone						▲			
**F13**	65.627	$C_{15}H_{10}O_5$	$[M-H]^-$	Trihydroxyisoflavone/flavone						▲	▲		
**F14**	60.827	$C_{17}H_{14}O_6$	$[M+H]^+$	Dihydroxy dimethoxyisoflavone/flavone						▲			
**F15**	57.460	$C_{17}H_{18}O_5$	$[M+H]^+$	Astraisoflavane isomer		▲			▲		▲		
**F16**	65.852	$C_{17}H_{16}O_6$	$[M+H]^+$	Dihydroxy methoxydihydroisoflavone/flavone						▲		▲	
**F17** ♥	73.328	$C_{17}H_{16}O_5$	$[M-H]^+$	Astraptercarpan		▲					▲		▲
**F18**	35.440	$C_{27}H_{32}O_{14}$	$[M-H]^-$	Naringin			▲	▲	▲	▲	▲	▲	▲
**F19**	34.635	$C_{28}H_{36}O_{13}$	$[M-H]^-$	Dihydrocalycosin pentose glucoside						▲			
**F20**	51.837	$C_{28}H_{34}O_{14}$	$[M-H]^-$	Astrapterocarpan pentose glucoside						▲			
**F21**	63.302	$C_{16}H_{14}O_5$	$[M+H]^+$	3,10-dihydroxy-9-methoxypterocarpan						▲			
**F22**	60.812	$C_{22}H_{22}O_{11}$	$[M-H]^-$	Pratensein glucoside/ 5,7',4'-trihydroxy -3'-methoxyisoflavone glucoside						▲			
**F23**	22.332	$C_{22}H_{22}O_{11}$	$[M-H]^-$	Pratensein glucoside/ 5,7',4'-trihydroxy -3'-methoxyisoflavone glucoside						▲			
				**Sum**	**3**	**5**	**4**	**3**	**5**	**17**	**7**	**7**	**6**

Note: t_R: Retention time; ❤ These constituents were identified by comparison with reference compounds; ※ New original constituents found *in vivo* after administration of ARTF. ▲ Detected.

2.2.2. Distribution of Metabolites

Table S3. The distribution of metabolites after administration of ARTF in 10 organs of rats.

No.	t_R (min)	Formula (M)	Ion	Identification	Heart	Liver	Spleen	Lungs	Kidneys	Stomach	Intestine	Colon	Thymus
**M5** △	21.008	$C_9H_8O_6S$	[M − H]$^-$	Hydroxycinnamic acid sulfate 1					▲				
**M12** △	54.552	$C_{11}H_{14}O_6S$	[M − H]$^-$	Methoxyeugenol sulfate 2						▲			
**M26**	47.512	$C_{16}H_{12}O_8S$	[M − H]$^-$	Calycosin sulfate 2		▲	▲	▲	▲	▲	▲	▲	▲
**M27**	51.168	$C_{16}H_{12}O_8S$	[M − H]$^-$	Calycosin sulfate isomer 1						▲	▲	▲	
**M32** △	36.133	$C_{22}H_{20}O_{11}$	[M − H]$^-$	Calycosin-3'-*O*-glucuronide	▲	▲	▲	▲	▲	▲	▲		▲
**M34** △	31.245	$C_{22}H_{24}O_{14}S$	[M − H]$^-$	Calycosin glucuronide sulfate 2					▲	▲	▲		
**M36**	57.743	$C_{16}H_{12}O_9S$	[M − H]$^-$	Hydroxycalycosin sulfate 2							▲		
**M37**	61.243	$C_{16}H_{12}O_9S$	[M − H]$^-$	Hydroxycalycosin sulfate 3					▲				
**M39** △	51.843	$C_{22}H_{20}O_{12}$	[M − H]$^-$	Hydroxycalycosin glucuronide 2					▲		▲		
**M40** △	39.042	$C_{23}H_{22}O_{12}$	[M − H]$^-$	Methoxycalycosin glucuronide						▲	▲		
**M41** △	33.123	$C_{23}H_{22}O_{15}S$	[M − H]$^-$	Methoxycalycosin glucuronide sulfate							▲		
**M43**	48.258	$C_{16}H_{14}O_8S$	[M − H]$^-$	Dihydrocalycosin sulfate 1							▲		
**M45**	45.652	$C_{16}H_{14}O_8S$	[M − H]$^-$	Dihydrocalycosin sulfate 3							▲		
**M46** △	38.098	$C_{22}H_{22}O_{11}$	[M − H]$^-$	Dihydrocalycosin glucuronide							▲		
**M50**	39.792	$C_{18}H_{18}O_8S$	[M − H]$^-$	Dimethyl dihydrocalycosin sulfate							▲	▲	
**M51** △	52.118	$C_{17}H_{16}O_9S$	[M − H]$^-$	Methoxy dihydrocalycosin sulfate 1						▲			
**M55**	19.342	$C_{16}H_{16}O_8S$	[M − H]$^-$	Tetrahydrocalycosin sulfate 2						▲		▲	
**M56**	21.443	$C_{16}H_{16}O_8S$	[M − H]$^-$	Tetrahydrocalycosin sulfate 3						▲	▲	▲	
**M57**	50.593	$C_{16}H_{16}O_8S$	[M − H]$^-$	Tetrahydrocalycosin sulfate 4		▲			▲	▲	▲	▲	
**M58**	67.630	$C_{16}H_{16}O_8S$	[M − H]$^-$	Tetrahydrocalycosin sulfate 5		▲			▲	▲	▲	▲	
**M61** △	54.027	$C_{22}H_{24}O_{11}$	[M − H]$^-$	Tetrahydrocalycosin glucuronide 2		▲			▲	▲	▲		
**M66**	64.843	$C_{16}H_{16}O_9S$	[M − H]$^-$	Hydroxy tetrahydrocalycosin sulfate 3		▲			▲				
**M70** △,★	45.733	$C_{18}H_{20}O_{10}S$	[M − H]$^-$	Dihydroxy dihydrocalycosin sulfate								▲	
**M71** △	56.593	$C_{24}H_{28}O_{12}$	[M − H]$^-$	Dimethyl hydroxy tetrahydrocalycosin glucuronide 1		▲				▲			
**M72** △	54.727	$C_{24}H_{28}O_{12}$	[M − H]$^-$	Dimethyl hydroxy tetrahydrocalycosin glucuronide 2		▲	▲	▲		▲	▲		

**M73** △	71.300	$C_{16}H_{12}O_7S$	$[M-H]^-$	Formononetin -7-*O*-sulfate						▲	▲		
**M74** △	49.357	$C_{22}H_{20}O_{10}$	$[M-H]^-$	Formononetin-7-*O*-glucuronide					▲	▲	▲		
**M76**	59.352	$C_{16}H_{16}O_7S$	$[M-H]^-$	Tetrahydroformononetin sulfate 2							▲	▲	
**M77**	59.860	$C_{16}H_{16}O_7S$	$[M-H]^-$	Tetrahydroformononetin sulfate 3							▲		
**M79** △	69.797	$C_{17}H_{16}O_8S$	$[M-H]^-$	Astrapterocarpan-3-*O*-sulfate						▲			
**M80** △	56.043	$C_{23}H_{24}O_{11}$	$[M-H]^-$	Astrapterocarpan-3-*O*-glucuronide	▲	▲		▲	▲	▲	▲		▲
**M81**	31.018	$C_{18}H_{18}O_6$	$[M-H]^-$	Methoxyastrapterocarpan								▲	
**M83** △	40.915	$C_{23}H_{24}O_{12}$	$[M-H]^-$	Hydroxyastraisoflavan glucuronide 2							▲		
**M84** △	43.618	$C_{23}H_{24}O_{12}$	$[M-H]^-$	Hydroxyastraisoflavan glucuronide 3							▲		
**M87** △	37.802	$C_{18}H_{20}O_5$	$[M-H]^-$	Methoxyastraisoflavan isomer						▲		▲	
**M88** △	34.368	$C_{19}H_{22}O_6$	$[M-H]^-$	Hydroxy dimethoxyastraisoflavan								▲	
**M90**	62.510	$C_{17}H_{18}O_8S$	$[M-H]^-$	Astraisoflavan-2'-*O*-sulfate		▲			▲				
**M91**	34.427	$C_{17}H_{18}O_8S$	$[M-H]^-$	Astraisoflavan sulfate isomer							▲	▲	
**M95**	49.543	$C_{17}H_{18}O_9S$	$[M-H]^-$	Hydroxyastraisoflavan sulfate 2						▲			
**M96**	44.008	$C_{17}H_{18}O_9S$	$[M-H]^-$	Hydroxyastraisoflavan sulfate 3		▲			▲	▲	▲	▲	
**M97** △	51.435	$C_{17}H_{18}O_9S$	$[M-H]^-$	Hydroxyastraisoflavan sulfate 4		▲			▲		▲	▲	
**M98** △	68.372	$C_{17}H_{18}O_9S$	$[M-H]^-$	Hydroxyastraisoflavan sulfate 5							▲	▲	
**M102** △	57.802	$C_{23}H_{26}O_{11}$	$[M-H]^-$	Astraisoflavan-7-*O*-glucuronide							▲		
**M103** △	57.002	$C_{23}H_{26}O_{11}$	$[M-H]^-$	Astraisoflavan-2'-*O*-glucuronide	▲	▲	▲	▲	▲	▲	▲	▲	▲
**M105** △★	52.510	$C_{23}H_{26}O_{14}S$	$[M-H]^-$	Astraisoflavan glucuronide sulfate 2							▲		
**M106** △	35.492	$C_{29}H_{36}O_{16}$	$[M-H]^-$	Astraisoflavan-7-*O*-glucoside-2'-*O*-glucuronide		▲		▲	▲	▲	▲		
**M107**	41.557	$C_{15}H_{10}O_7S$	$[M-H]^-$	Daidzein-4'-*O*-sulfate		▲	▲		▲	▲	▲	▲	
**M112** △	44.292	$C_{15}H_{12}O_7S$	$[M-H]^-$	Dihydrodaidzein sulfate							▲		
**M113**	53.852	$C_{15}H_{14}O_7S$	$[M-H]^-$	Tetrahydrodaidzein sulfate 1					▲				
**M115**	57.002	$C_{15}H_{14}O_7S$	$[M-H]^-$	Tetrahydrodaidzein sulfate 3							▲		
**M120**	57.918	$C_{15}H_{10}O_8S$	$[M-H]^-$	Genistein sulfate 2							▲		
**M121**	37.233	$C_{15}H_{10}O_8S$	$[M-H]^-$	Genistein sulfate 3							▲		
**M126**	44.750	$C_{15}H_{14}O_5$	$[M-H]^-$	Tetrahydrogenistein						▲			
**M127**	63.985	$C_{15}H_{14}O_8S$	$[M-H]^-$	Tetrahydrogenistein sulfate 1		▲			▲		▲	▲	
**M128**	65.410	$C_{15}H_{14}O_8S$	$[M-H]^-$	Tetrahydrogenistein sulfate 2						▲			

**M130** △,★	39.850	$C_{21}H_{24}O_{13}S$	$[M-H]^-$	Tetrahydrogenistin sulfate						▲		
**M132**	56.152	$C_{15}H_{14}O_6S$	$[M-H]^-$	Equol sulfate 1	▲			▲	▲	▲	▲	▲
**M133**	58.943	$C_{15}H_{14}O_6S$	$[M-H]^-$	Equol sulfate 2	▲			▲	▲	▲	▲	▲
**M136** △,★	32.293	$C_{21}H_{22}O_{12}S$	$[M-H]^-$	Equol glucuronide sulfate 1						▲		
**M137** △,★	28.292	$C_{21}H_{22}O_{12}S$	$[M-H]^-$	Equol glucuronide sulfate 2						▲		
**M138** △	37.317	$C_{21}H_{22}O_{12}S$	$[M-H]^-$	Equol glucuronide sulfate 3						▲		
**M139** △	57.977	$C_{15}H_{16}O_6S$	$[M-H]^-$	Dihydroequol sulfate						▲		
**M145** △	61.012	$C_{22}H_{20}O_{11}$	$[M-H]^-$	Calycosin-7-*O*-glucuronide					▲			
**M146** △	41.930	$C_{22}H_{20}O_{15}S$	$[M-H]^-$	Pratensein glucuronide sulfate						▲		
**M147** △	69.988	$C_{17}H_{18}O_8S$	$[M-H]^-$	Astraisoflavane sulfate					▲			

Note: t_R: Retention time. △ New metabolite found i*n vivo* after administration of ARTF; ★ Potential New compound by retrieving information from Sci Finder database; ▲ Detected. No compounds were detected in the brain.

2.2.3. Pharmacological Effect of in vivo Compounds of ARTF

Table S4. Summary of ARTF-related pharmacological effect of Compounds in vivo.

**Compound in vivo**			**ARTF-related pharmacological effect**					
**Type**	**Name**	**NO.**	**Cardiovascular Protective**	**Neuro-protective**	**Anti-inflammatory**	**Anti-tumor**	**Hepatic Protective**	**Estrogen Receptor activation**
**Original constituents**	Calycosin	**F1**	✓[39]	✓[40]	✓[41]	✓[42]	✓[43]	✓[48]
	Calycosin-7-*O*-glucoside	**F4**	✓[44]	✓[45]	✓[46]	✓[47]		✓[48]
	Ononin	**F5**		✓[40,48]	✓[49]			✓[48]
	Formononetin	**F6**	✓[39]	✓[40]	✓[50]	✓[51]	✓[52]	✓[48]
	Daidzein	**F11**	✓[53]	✓[48,54]	✓[50]	✓[55]	✓[56]	✓[48]
	Naringin	**F18**	✓[57]	✓[58]	✓[58]	✓[59]	✓[60]	✓[61]
**Metabolites**	Daidzein-4′-*O*-sulfate	**M107**						✓[62]
	Daidzein-7-*O*-sulfate	**M108**						✓[62]

Daidzein-7-*O*-glucuronide	**M110**	✔ [63]					✔ [64]
Daidzein-4′-*O*-glucuronide							✔ [65]
Genistein	**M118**	✔ [53]	✔ [54]	✔ [50]	✔ [65]	✔ [66]	✔ [48]
Genistein-7-*O*-sulfate	**One of M119-M121**	✔ [67]			✔ [68]		✔ [62]
Genistein-4′-*O*-sulfate	**One of M119-M121**	✔ [69]			✔ [68]		✔ [62,64]
Equol-7-*O*-sulfate	**M132 or M133**						✔ [62]

2.2.4. The Constituents of ARTF

Table S5. 69 kinds of constituents identified in ARTF.

NO.	t_R (min)	Formula (M)	Ion	Meas. (*m*/*z*)	Pred. (*m*/*z*)	Diff (ppm)	DBE	Identification
**1**	54.277	$C_{15}H_{10}O_4$	$[M-H]^-$	253.0505	253.0506	−0.40	11	Daidzein
**2**	55.693	$C_{15}H_{12}O_4$	$[M-H]^-$	255.0683	255.0663	7.84	10	4,4',6'-trihydroxychalcone
**3**	53.152	$C_{15}H_{10}O_4$	$[M+H]^+$	255.0687	255.0652	13.72	11	Dihydroxyisoflavone
**4**	71.335	$C_{15}H_{12}O_4$	$[M+H]^+$	257.0802	257.0808	−2.33	10	Trihydroxychalcone
**5**	28.777	$C_{15}H_{12}O_4$	$[M+H]^+$	257.0817	257.0808	3.50	10	4,2,6'-trihydroxychalcone
**6**	71.802	$C_{16}H_{12}O_4$	$[M-H]^-$	267.0660	267.0663	−1.12	11	Formononetin
**7**	31.360	$C_{15}H_{10}O_5$	$[M-H]^-$	269.0450	269.0455	−1.86	11	Trihydroxyisoflavone
**8**	55.118	$C_{15}H_{10}O_5$	$[M-H]^-$	269.0459	269.0455	1.49	11	Trihydroxyisoflavone
**9**	39.727	$C_{15}H_{10}O_5$	$[M-H]^-$	269.0472	269.0455	6.32	11	Trihydroxyisoflavone
**10**	66.015	$C_{15}H_{10}O_5$	$[M-H]^-$	269.0454	269.0455	−0.37	11	Trihydroxyisoflavone
**11**	41.002	$C_{15}H_{12}O_5$	$[M-H]^-$	271.0609	271.0612	−1.11	10	Dihydrotrihydroxyisoflavone
**12**	64.760	$C_{15}H_{12}O_5$	$[M-H]^-$	271.0622	271.0612	3.69	10	Dihrdrogenistein
**13**	62.610	$C_{16}H_{14}O_4$	$[M+H]^+$	271.0955	271.0965	−1.00	10	Dihydroformononetin
**14**	45.168	$C_{15}H_{14}O_5$	$[M-H]^-$	273.0771	273.0768	1.10	9	Tetrahydrotrihydroxyisoflavone
**15**	58.635	$C_{16}H_{12}O_5$	$[M-H]^-$	283.0615	283.0612	1.06	11	Calycosin
**16**	56.765	$C_{16}H_{12}O_5$	$[M-H]^-$	283.0610	283.0612	−0.71	11	Calycosin isomer
**17**	78.035	$C_{16}H_{12}O_5$	$[M+H]^+$	285.0779	285.0758	7.37	11	Calycosin isomer
**18**	50.185	$C_{16}H_{12}O_5$	$[M+H]^+$	285.0782	285.0758	8.42	11	Calycosin isomer
**19**	63.393	$C_{16}H_{14}O_5$	$[M+H]^+$	287.0927	287.0914	4.53	10	Dihydrocalycosin isomer
**20**	37.010	$C_{16}H_{14}O_5$	$[M+H]^+$	287.0938	287.0914	8.36	10	Dihydrocalycosin isomer
**21**	37.635	$C_{16}H_{12}O_6$	$[M-H]^-$	299.0538	299.0561	−7.69	11	Pratensein/Rhamnocitrin /5,7,4'-trihydroxy-3'-methoxyisoflavone
**22**	36.718	$C_{16}H_{12}O_6$	$[M-H]^-$	299.0572	299.0561	3.68	11	Pratensein/Rhamnocitrin /5,7,4'-trihydroxy-3'-methoxyisoflavone
**23**	67.207	$C_{16}H_{12}O_6$	$[M-H]^-$	299.0560	299.0561	−0.33	11	Pratensein/Rhamnocitrin /5,7,4'-trihydroxy-3'-methoxyisoflavone
**24**	68.332	$C_{16}H_{12}O_6$	$[M-H]^-$	299.0557	299.0561	−1.34	11	Pratensein/Rhamnocitrin /5,7,4'-trihydroxy-3'-methoxyisoflavone
**25**	73.685	$C_{17}H_{16}O_5$	$[M+H]^+$	301.1074	301.1071	1.00	10	Astrapterocarpan
**26**	57.818	$C_{17}H_{18}O_5$	$[M+H]^+$	303.1211	303.1227	−5.28	9	Astraisoflavane isomer
**27**	74.077	$C_{17}H_{18}O_5$	$[M+H]^+$	303.1241	303.1227	4.62	9	Astraisoflavane
**28**	61.177	$C_{17}H_{14}O_6$	$[M+H]^+$	315.0876	315.0863	4.13	11	Dihrdroxymethoxyisoflavone
**29**	28.485	$C_{17}H_{14}O_6$	$[M+H]^+$	315.0860	315.0863	−0.95	11	Dihrdroxymethoxyisoflavone

**30**	51.519	$C_{18}H_{18}O_5$	$[M + H]^+$	315.1259	315.1227	10.15	10	Trimethoxypterocarpan
**31**	65.852	$C_{17}H_{16}O_6$	$[M + H]^+$	317.1040	317.1020	6.31	10	Hydroxyastrapterocarpan
**32**	42.027	$C_{17}H_{18}O_6$	$[M - H]^-$	317.1029	317.1031	−0.63	9	Dihydro-droxyastrapterocarpan
**33**	28.850	$C_{21}H_{22}O_9$	$[M - H]^-$	417.1219	417.1191	6.71	11	Trihydroxychalcone glucoside
**34**	36.777	$C_{21}H_{22}O_{10}$	$[M - H]^-$	433.1112	433.1140	−6.46	11	Dihydro-trihydroxyisoflavone glucoside
**35**	25.985	$C_{21}H_{24}O_{10}$	$[M - H]^-$	435.1284	435.1297	−2.99	10	Tetrahydro-trihydroxyisoflavone glucoside
**36**	23.552	$C_{21}H_{24}O_{10}$	$[M - H]^-$	435.1318	435.1297	4.83	10	Tetrahydro-trihydroxyisoflavone glucoside
**37**	36.660	$C_{22}H_{24}O_{10}$	$[M - H]^-$	447.1315	447.1297	4.03	11	Dihydrocalycosin glucoside
**38**	21.293	$C_{21}H_{22}O_{11}$	$[M - H]^-$	449.1073	449.1089	−3.56	11	Dihydro-tetrahydroxyisoflavone glucoside
**39**	30.343	$C_{21}H_{22}O_{11}$	$[M - H]^-$	449.1100	449.1089	2.45	11	Dihydro-tetrahydroxyisoflavone glucoside
**40**	53.170	$C_{22}H_{26}O_{10}$	$[M - H]^-$	449.1446	449.1453	−1.56	10	Tetrahydrocalycosin glucoside
**41**	23.435	$C_{21}H_{20}O_9$	$[M + HCOO]^-$	461.1105	461.1089	3.47	12	Daidzein glucoside
**42**	25.768	$C_{22}H_{22}O_{11}$	$[M - H]^-$	461.1055	461.1089	−7.37	12	Pratensein glucoside/ 5,7',4'-trihydroxy -3′-methoxyisoflavone glucoside
**43**	22.377	$C_{22}H_{22}O_{11}$	$[M - H]^-$	461.1112	461.1089	4.99	12	Pratensein glucoside/ 5,7',4' -trihydroxy-3′-methoxyisoflavone glucoside
**44**	60.710	$C_{22}H_{22}O_{11}$	$[M - H]^-$	461.1114	461.1089	5.42	12	Pratensein glucoside/ 5,7',4'-trihydroxy -3′-methoxyisoflavone glucoside
**45**	37.468	$C_{22}H_{22}O_{11}$	$[M + HCOO]^-$	507.1153	507.1144	1.77	12	Pratensein glucoside/ 5,7',4'-trihydroxy-3′-methoxyisoflavone glucoside
**46**	33.710	$C_{22}H_{22}O_{11}$	$[M + HCOO]^-$	507.1183	507.1144	7.69	12	Pratensein glucoside/ 5,7',4'-trihydroxy -3′-methoxyisoflavone glucoside
**47**	57.135	$C_{22}H_{22}O_{11}$	$[M + H]^+$	463.1261	463.1235	5.61	12	Pratensein glucoside/ 5,7',4'-trihydroxy-3′-methoxyisoflavone glucoside
**48**	50.842	$C_{23}H_{24}O_{10}$	$[M + H]^+$	461.1444	461.1442	0.43	12	Hydroxy dimethoxyisoflavone glucoside
**49**	29.818	$C_{21}H_{20}O_{12}$	$[M - H]^-$	463.0876	463.0882	−1.30	12	Isoquercetin
**50**	30.402	$C_{21}H_{20}O_{12}$	$[M - H]^-$	463.0899	463.0882	3.67	12	Isoquercetin isomer
**51**	37.185	$C_{22}H_{24}O_{11}$	$[M - H]^-$	463.1252	463.1246	1.30	11	Dihydropratensein glucoside
**52**	39.493	$C_{22}H_{24}O_{11}$	$[M - H]^-$	463.1264	463.1246	3.89	11	Dihydropratensein glucoside isomer
**53**	56.977	$C_{23}H_{28}O_{10}$	$[M - H]^-$	463.1618	463.1610	1.73	10	Astraisoflavane-7-*O*-glucoside
**54**	47.577	$C_{22}H_{22}O_9$	$[M + HCOO]^-$	475.1262	475.1246	3.37	12	Ononin
**55**	31.985	$C_{21}H_{20}O_{10}$	$[M + HCOO]^-$	477.1055	477.1038	3.56	12	Trihydroxyisoflavone glucoside
**56**	28.660	$C_{23}H_{24}O_{11}$	$[M + H]^+$	477.1364	477.1391	−5.66	12	Dihydroxy dimethoxyisoflavone glucoside
**57**	50.710	$C_{21}H_{18}O_{11}$	$[M + H]^+$	489.1425	489.1391	6.95	13	Calycosin-7-*O*-glucoside-6''-*O*-acetate isomer
**58**	27.610	$C_{22}H_{22}O_{10}$	$[M + HCOO]^-$	491.1215	491.1195	4.07	12	Calycosin-7-*O*-glucoside
**59**	24.985	$C_{22}H_{22}O_{10}$	$[M + HCOO]^-$	491.1215	491.1195	4.07	12	Calycosin-7-*O*-glucoside isomer
**60**	60.943	$C_{22}H_{22}O_{10}$	$[M + HCOO]^-$	491.1233	491.1195	7.74	12	Calycosin-7-*O*-glucoside isomer
**61**	43.003	$C_{22}H_{22}O_{10}$	$[M - H]^-$	445.1154	445.1140	3.15	12	Calycosin-7-*O*-glucoside isomer
**62**	46.835	$C_{24}H_{30}O_{11}$	$[M - H]^-$	493.1721	493.1715	1.22	10	Dihydro methoxyastrapterocarpan glucoside
**63**	54.433	$C_{23}H_{26}O_{10}$	$[M + HCOO]^-$	507.1521	507.1508	2.56	11	Astrapterocarpan-3-*O*-glucoside

**64**	32.102	$C_{23}H_{28}O_{11}$	[M + HCOO]$^-$	525.1647	525.1614	6.28	10	Hydroisoflavane glucoside
**65**	35.252	$C_{27}H_{32}O_{14}$	[M − H]$^-$	579.1748	579.1719	5.01	12	Naringin
**66**	34.293	$C_{28}H_{36}O_{13}$	[M − H]$^-$	579.2103	579.2083	3.45	11	Dihydrocalycosin+Pentose+Hexose
**67**	51.343	$C_{28}H_{34}O_{14}$	[M − H]$^-$	593.1899	593.1876	3.88	12	Astrapterocarpan+Pentose+Hexose
**68**	46.110	$C_{29}H_{38}O_{15}$	[M − H]$^-$	625.2158	625.2138	3.20	11	Astraisoflavane diglucoside
**69**	34.002	$C_{29}H_{38}O_{15}$	[M + HCOO]$^-$	671.2214	671.2193	3.13	11	Astraisoflavan-7,3'-di-*O*-glucoside

Note: t_R: retention time; Meas.: measured; Pred.: predicted; Diff: difference; DBE: double bone equivalents.

2.3. The EICs of Original Constituents and Metabolites in Plasma and Feces (Figure S1–Figure S4)

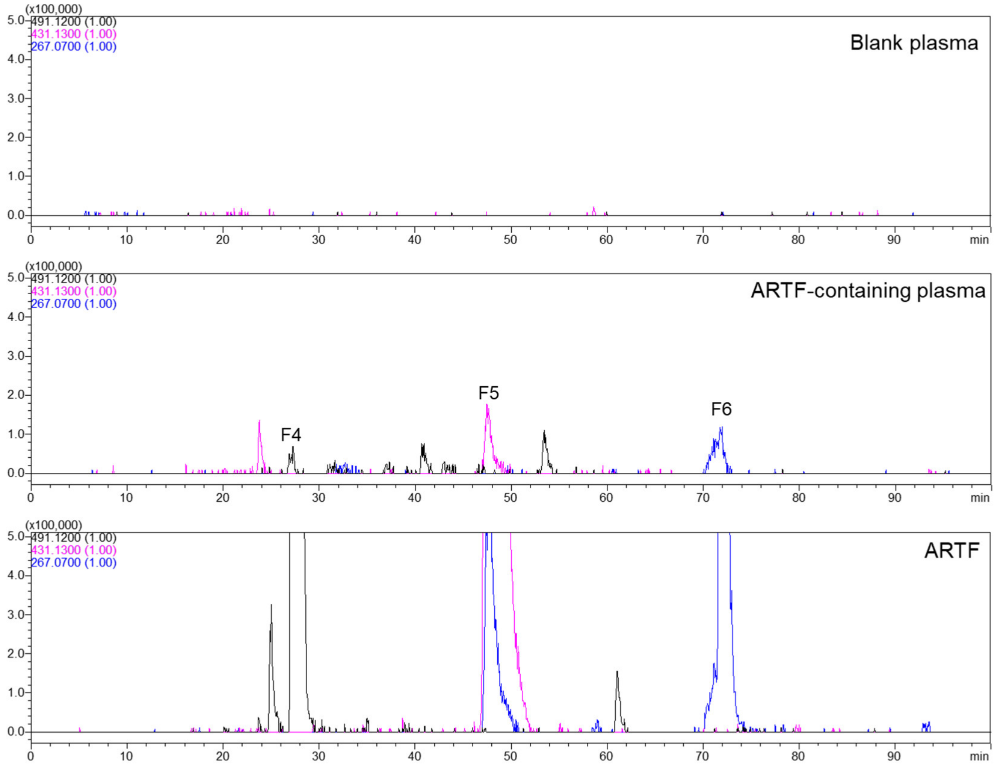

Figure S1. The EICs of original constituents in rat plasma after administration of ARTF.

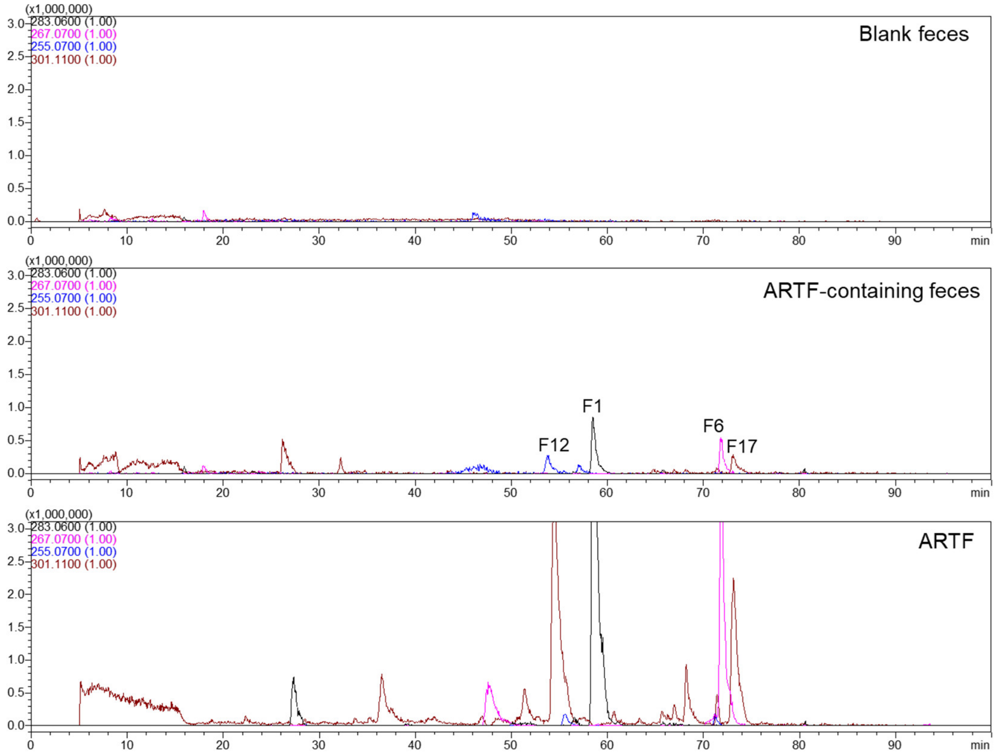

Figure S2. The EICs of original constituents in rat feces after administration of ARTF.

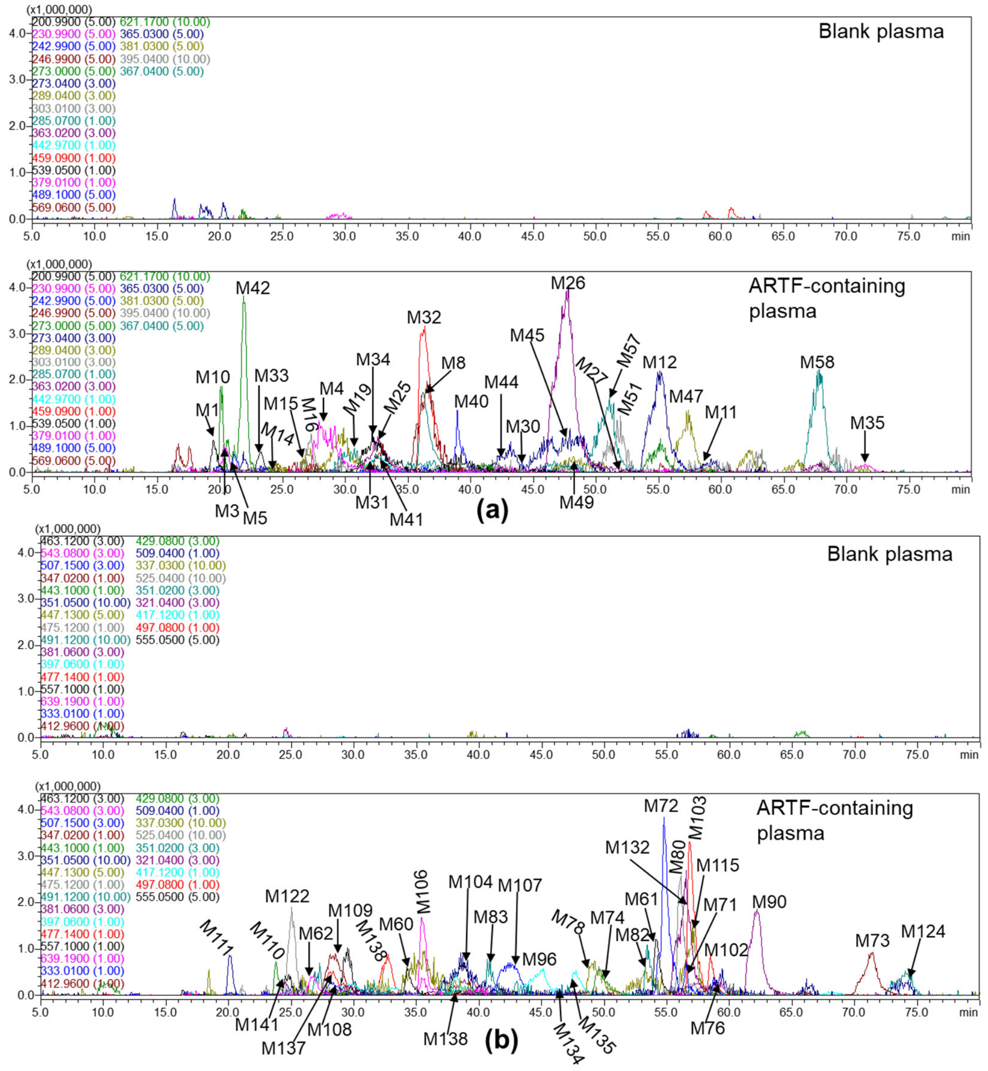

Figure S3. The EICs of metabolites in rat plasma after administration of ARTF.

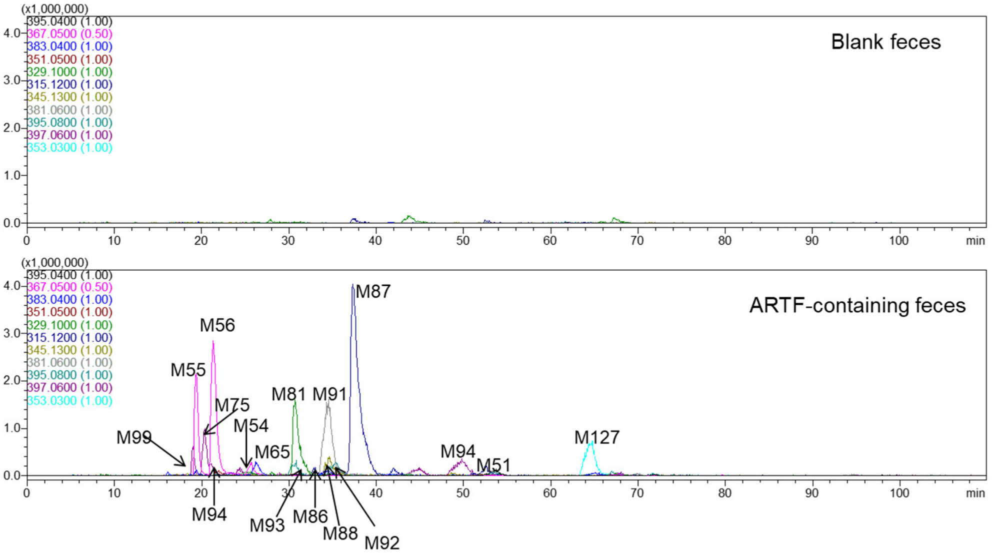

Figure S4. The EICs of metabolites in rat feces after administration of ARTF.

2.4. The MS² and MS³ Spectra of Detected New Compounds (Figure S5–Figure S16)

Event#: 5 MS/MS(E-) Ret. Time : 26.975 Scan# : 2669 Precursor : 543.0792 Cutoff : 150

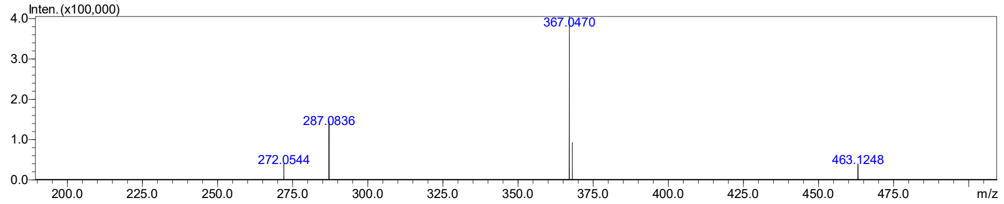

Figure S5. MS² spectrum of **M62.**

Event#: 5 MS/MS(E-) Ret. Time : 32.350 Scan# : 3272 Precursor : 639.1607 Cutoff : 176

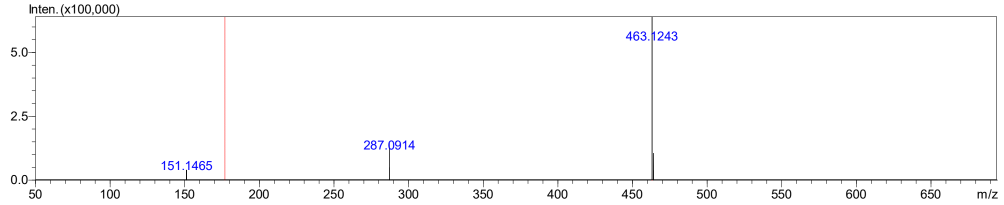

Figure S6. MS² spectrum of **M63.**

Event#: 5 MS/MS(E-) Ret. Time : 27.675 Scan# : 2792 Precursor : 463.0017 Cutoff : 128

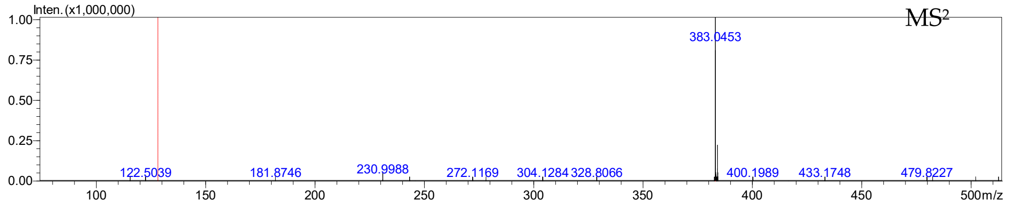

Event#: 6 MS3(E-) Ret. Time : 27.675 Scan# : 2793 Precursor : 383.0453 Cutoff : 106

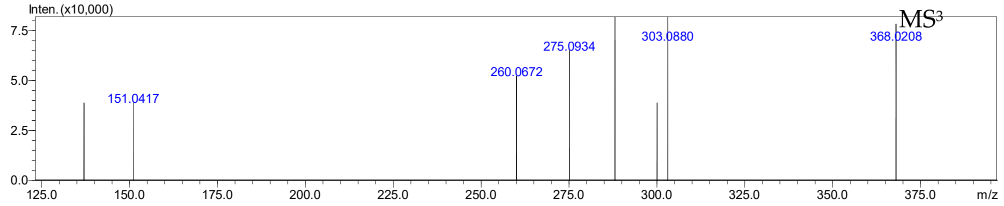

Figure S7. MS² and MS³ spectrum of **M69.**

Event#: 5 MS/MS(E-) Ret. Time : 45.733 Scan# : 4646 Precursor : 427.0687 Cutoff : 118

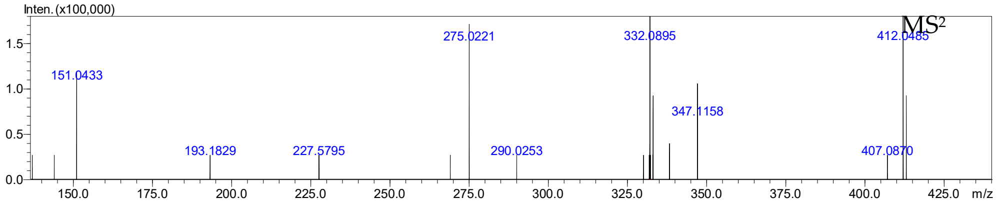

Event#: 6 MS3(E-) Ret. Time : 45.733 Scan# : 4647 Precursor : 332.0910 Cutoff : 91

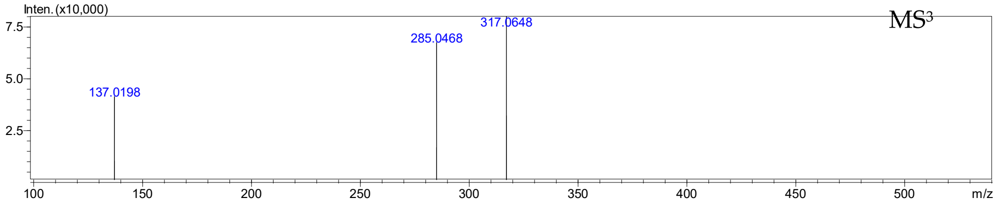

Figure S8. MS² and MS³ spectrum of **M70.**

Event#: 5 MS/MS(E-) Ret. Time : 38.250 Scan# : 3825 Precursor : 557.0997 Cutoff : 154

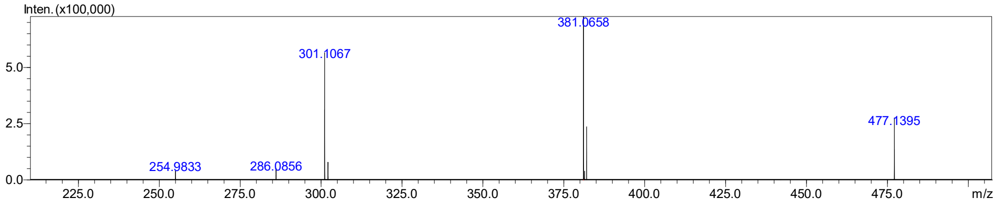

Figure S9. MS² spectrum of **M104.**

Event#: 5 MS/MS(E-) Ret. Time : 52.51 Scan# : 5291 Precursor : 557.0997 Cutoff : 154

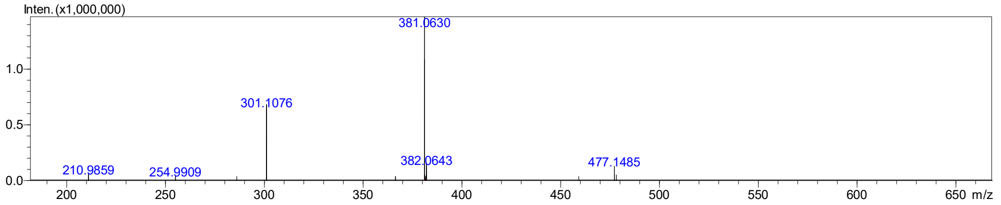

Figure S10. MS² spectrum of M105.

Event#: 5 MS/MS(E-) Ret. Time : 49.090 Scan# : 4990 Precursor : 485.0787 Cutoff : 134

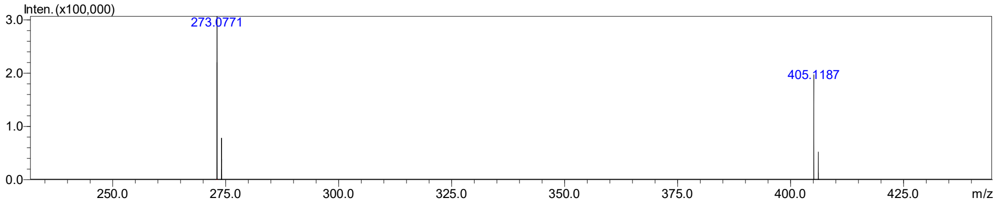

Figure S11. MS² spectrum of M129.

Event#: 5 MS/MS(E-) Ret. Time : 39.850 Scan# : 4042 Precursor : 515.0887 Cutoff : 142

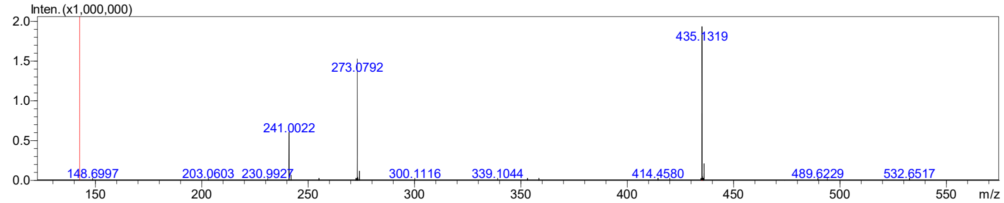

Figure S12. MS^2 spectrum of M130.

Event#: 5 MS/MS(E-) Ret. Time : 32.292 Scan# : 3214 Precursor : 497.0786 Cutoff : 137

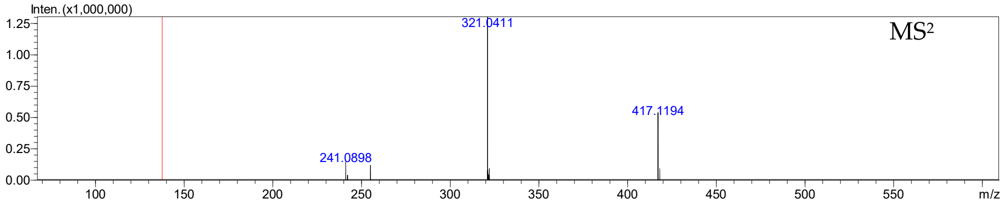

Event#: 6 MS3(E-) Ret. Time : 32.292 Scan# : 3215 Precursor : 321.0411 Cutoff : 88

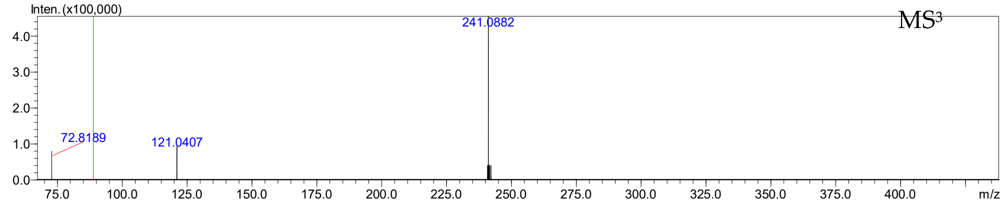

Figure S13. MS^2 and MS^3 spectrum of **M136.**

Event#: 5 MS/MS(E-) Ret. Time : 28.292 Scan# : 2804 Precursor : 497.0806 Cutoff : 137

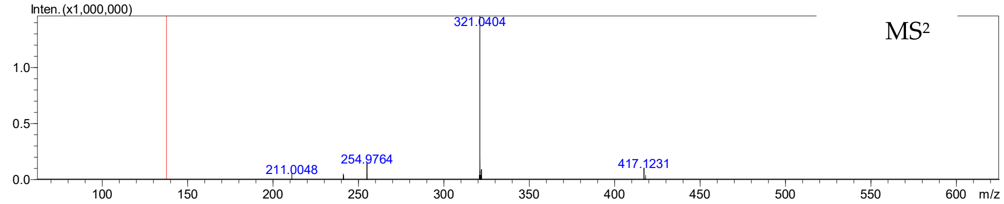

Event#: 6 MS3(E-) Ret. Time : 28.292 Scan# : 2805 Precursor : 321.0404 Cutoff : 88

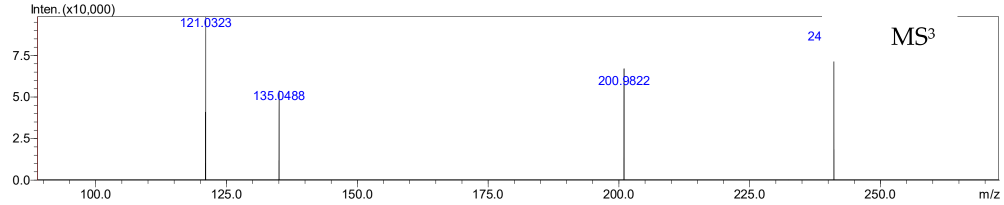

Figure S14. MS^2 and MS^3 spectrum of **M137.**

Event#: 5 MS/MS(E-) Ret. Time : 26.300 Scan# : 2627 Precursor : 625.1440 Cutoff : 173

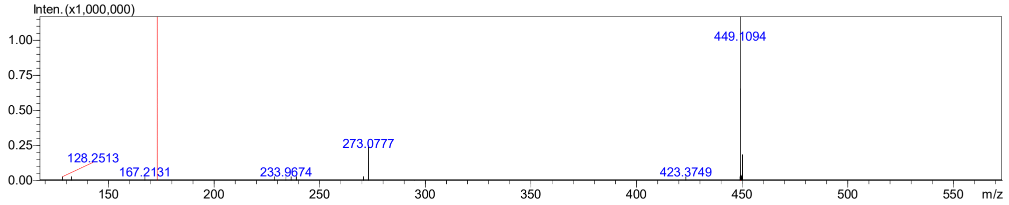

Figure S15. MS^2 spectrum of M142.

Event#: 5 MS/MS(E-) Ret. Time : 26.867 Scan# : 2863 Precursor : 625.1440 Cutoff : 83

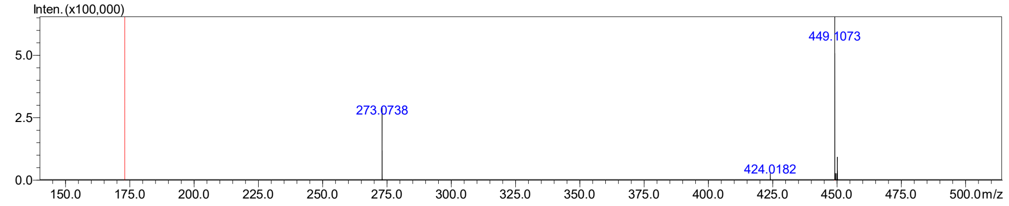

Figure S16. MS^2 spectrum of M143.

2.5. The EICs of Original Constituents and Metabolites Distributed in Organs (Figure S17–Figure S25)

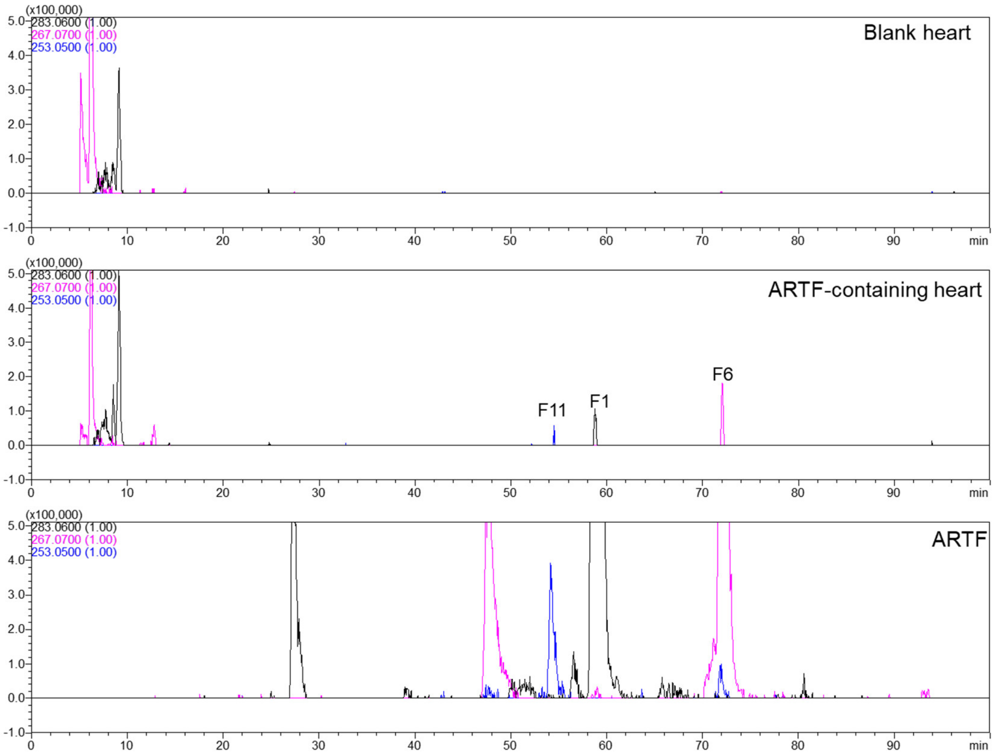

(a) Original constituents in heart

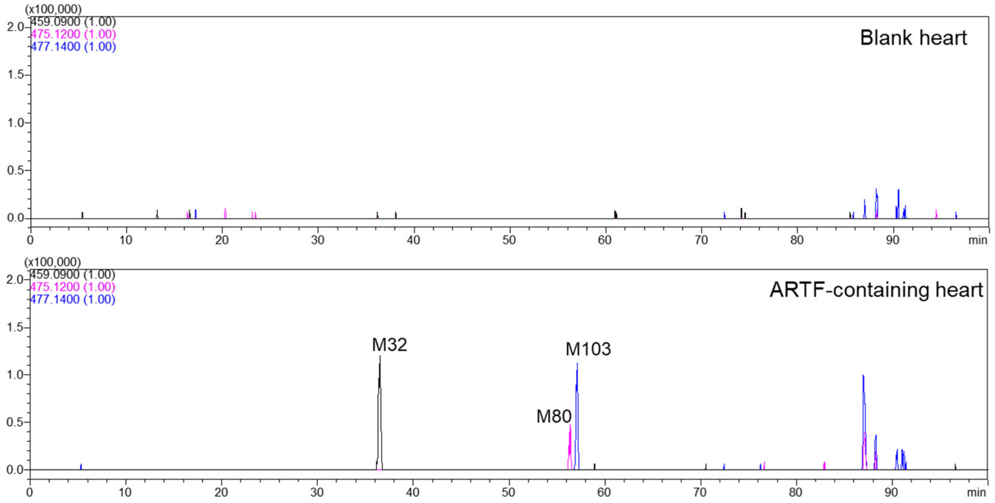

(b) Metabolites in heart

Figure S17. The EICs of original constituents and metabolites distributed in heart tissue of rat after administration of ARTF. (a: original constituents; b: metabolites).

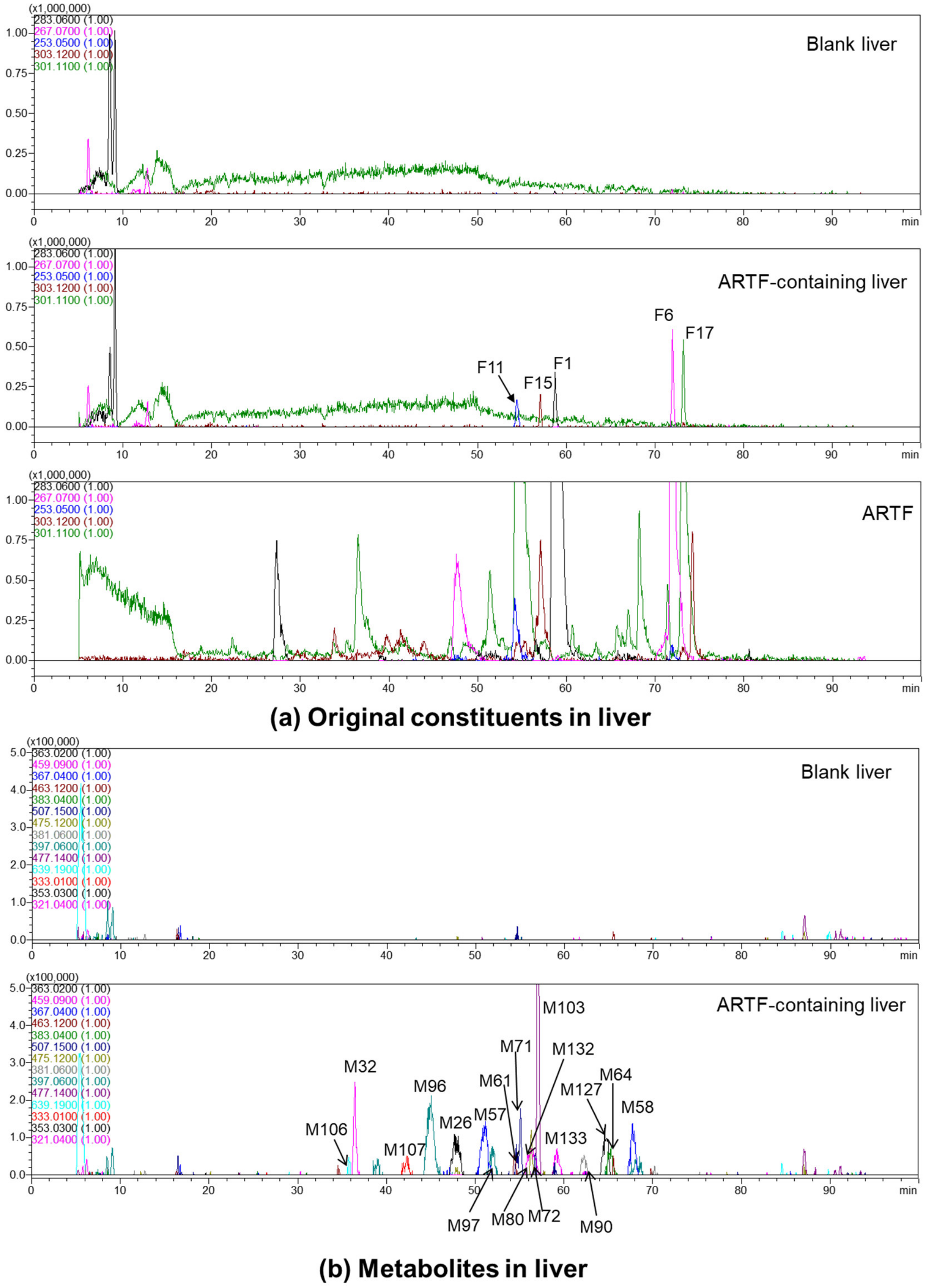

Figure S18. The EICs of original constituents and metabolites distributed in liver tissue of rat after administration of ARTF. (a: original constituents; b: metabolites).

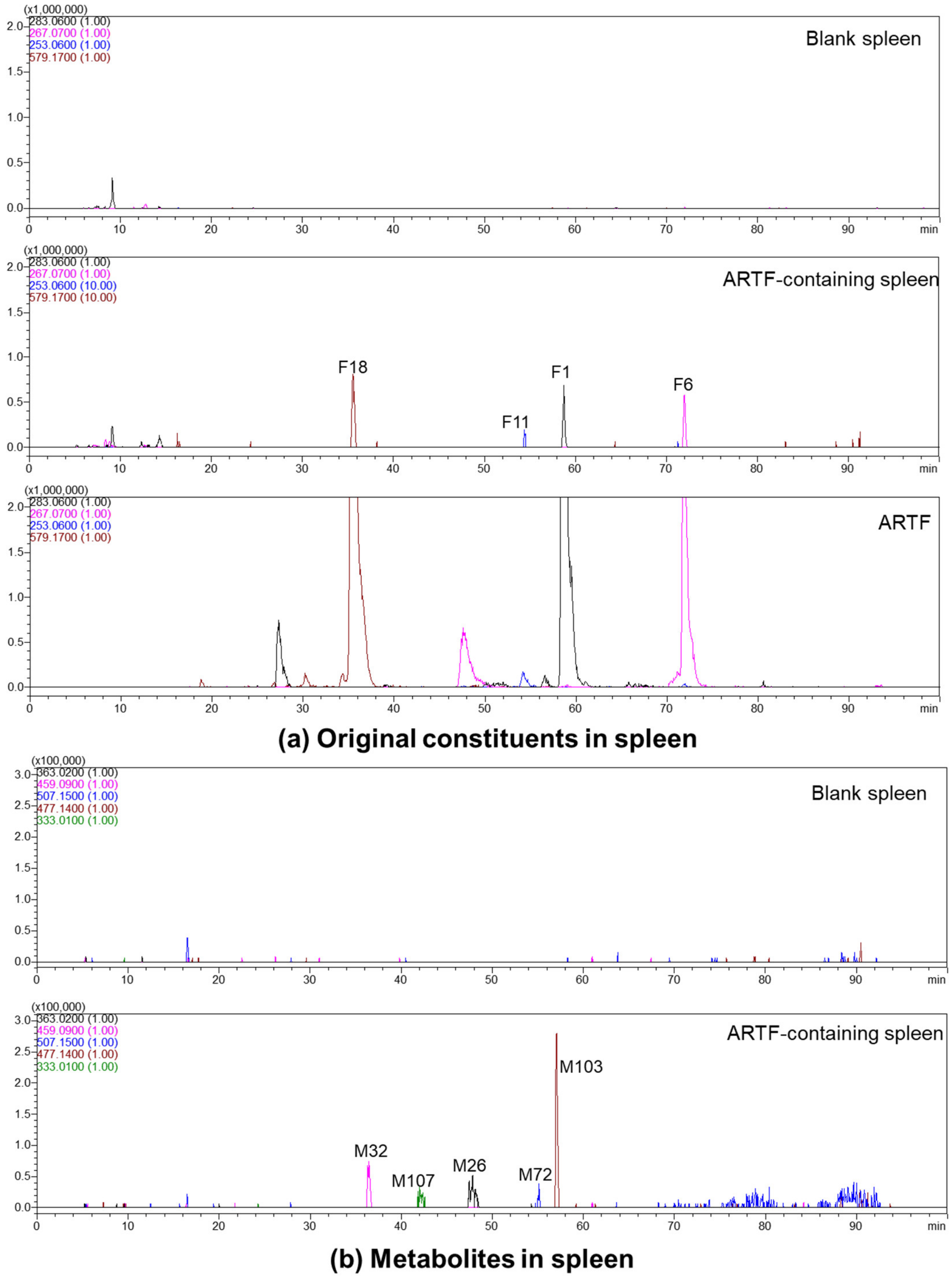

Figure S19. The EICs of original constituents and metabolites distributed in spleen tissue of rat after administration of ARTF. (a: original constituents; b: metabolites).

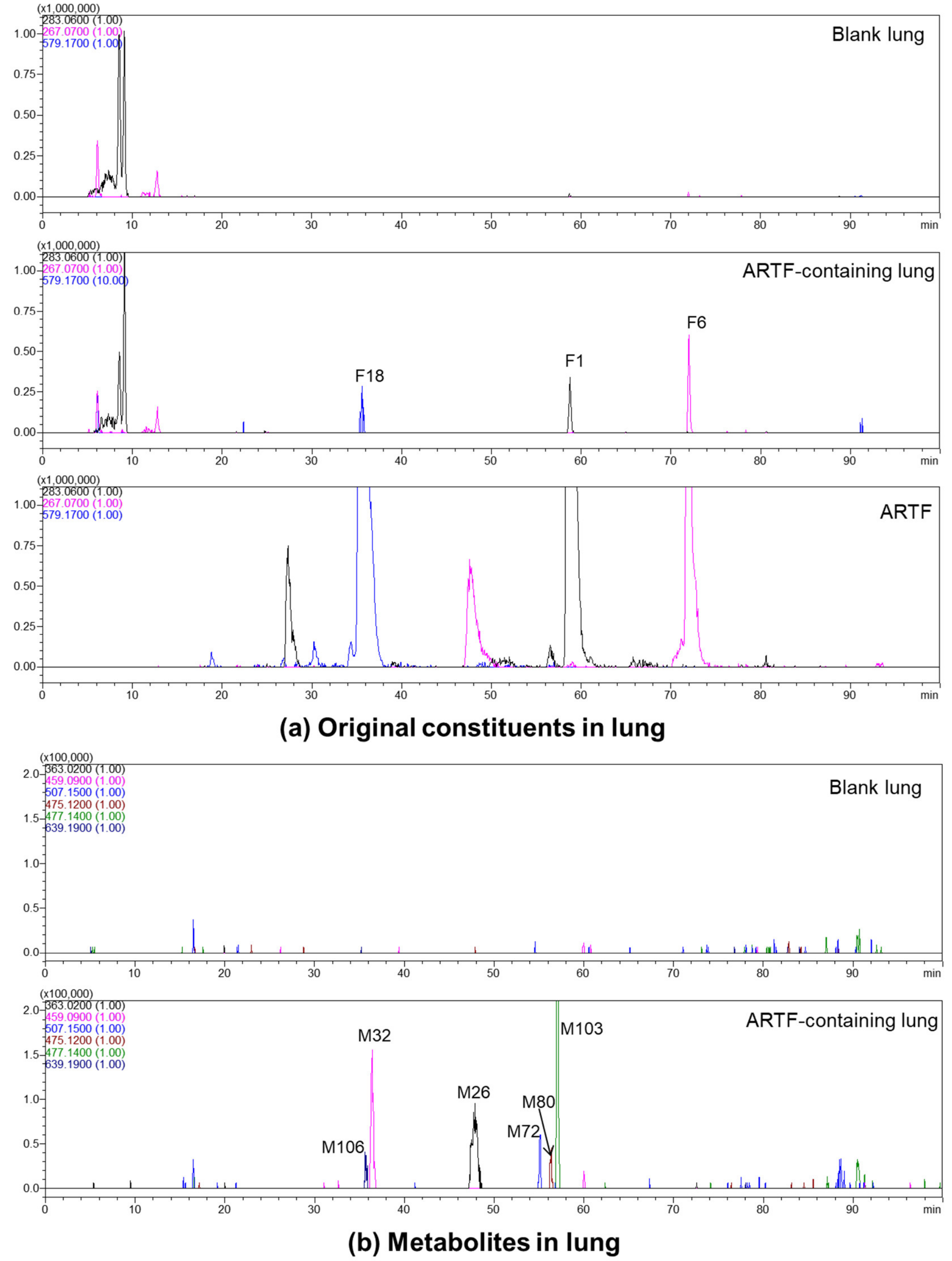

Figure S20. The EICs of original constituents and metabolites distributed in lung tissue of rat after administration of ARTF. (a: original constituents; b: metabolites).

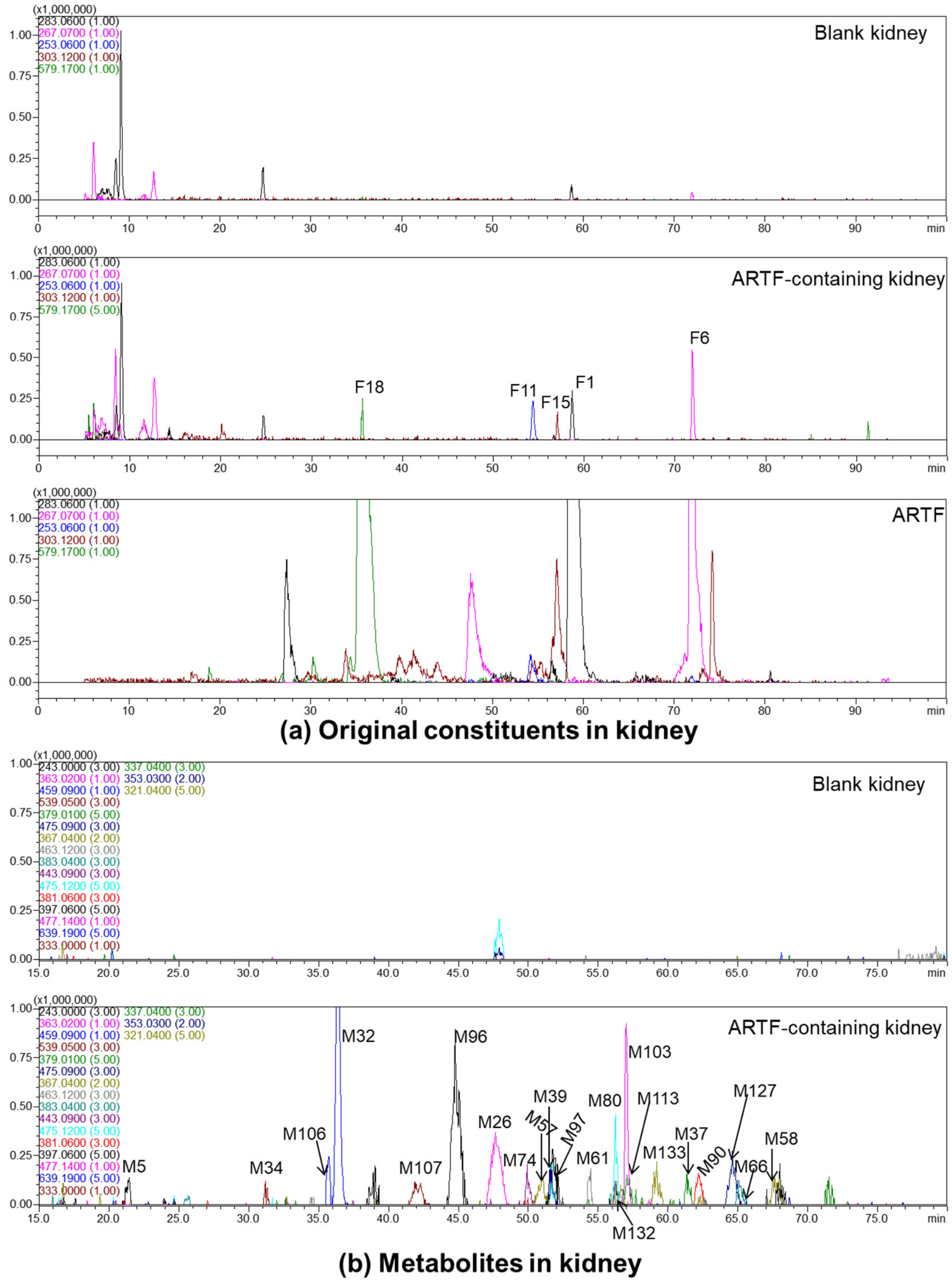

Figure S21. The EICs of original constituents and metabolites distributed in kidney tissue of rat after administration of ARTF. (a: original constituents; b: metabolites).

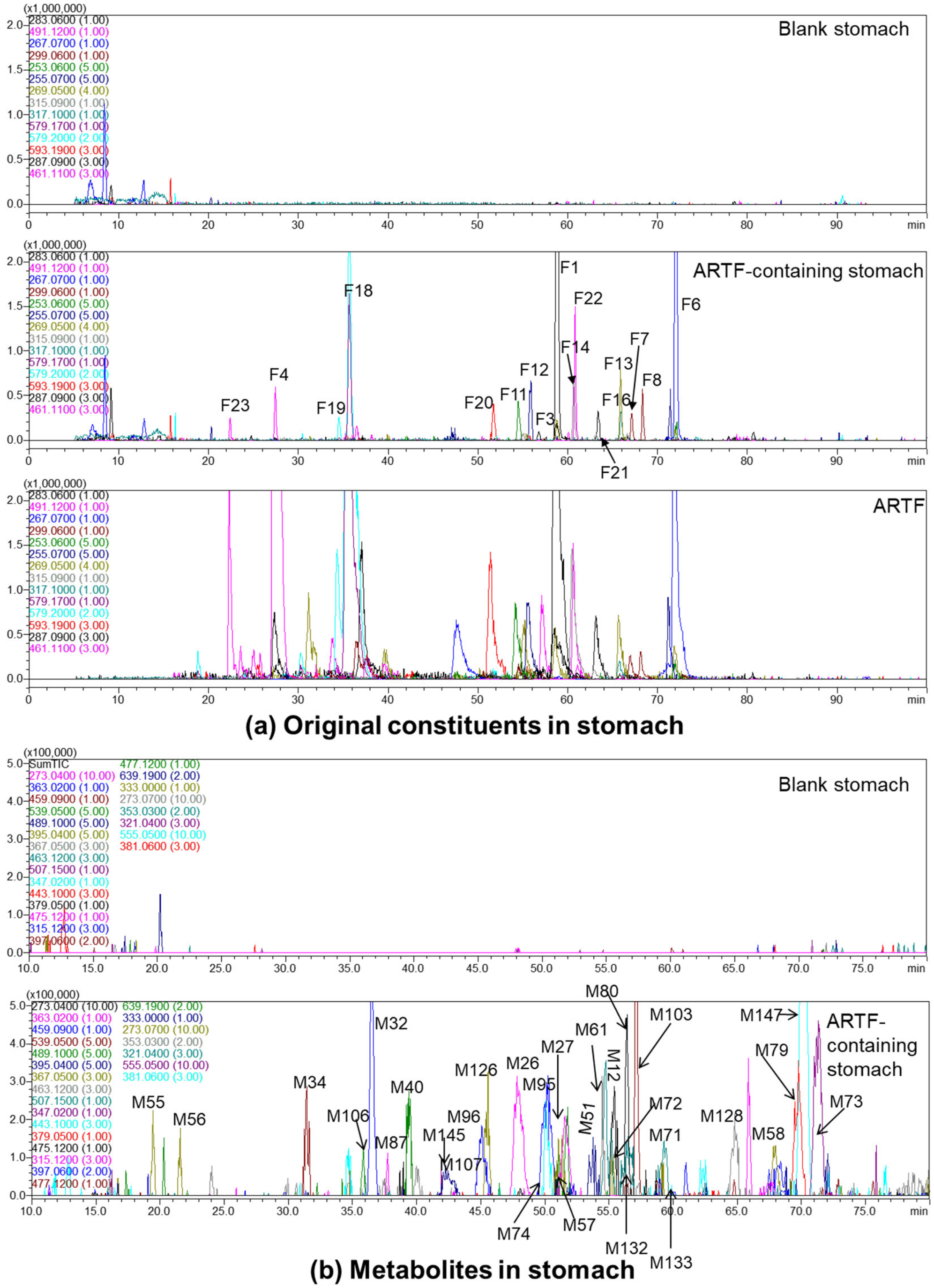

Figure S22. The EICs of original constituents and metabolites distributed in stomach tissue of rat after administration of ARTF. (a: original constituents; b: metabolites).

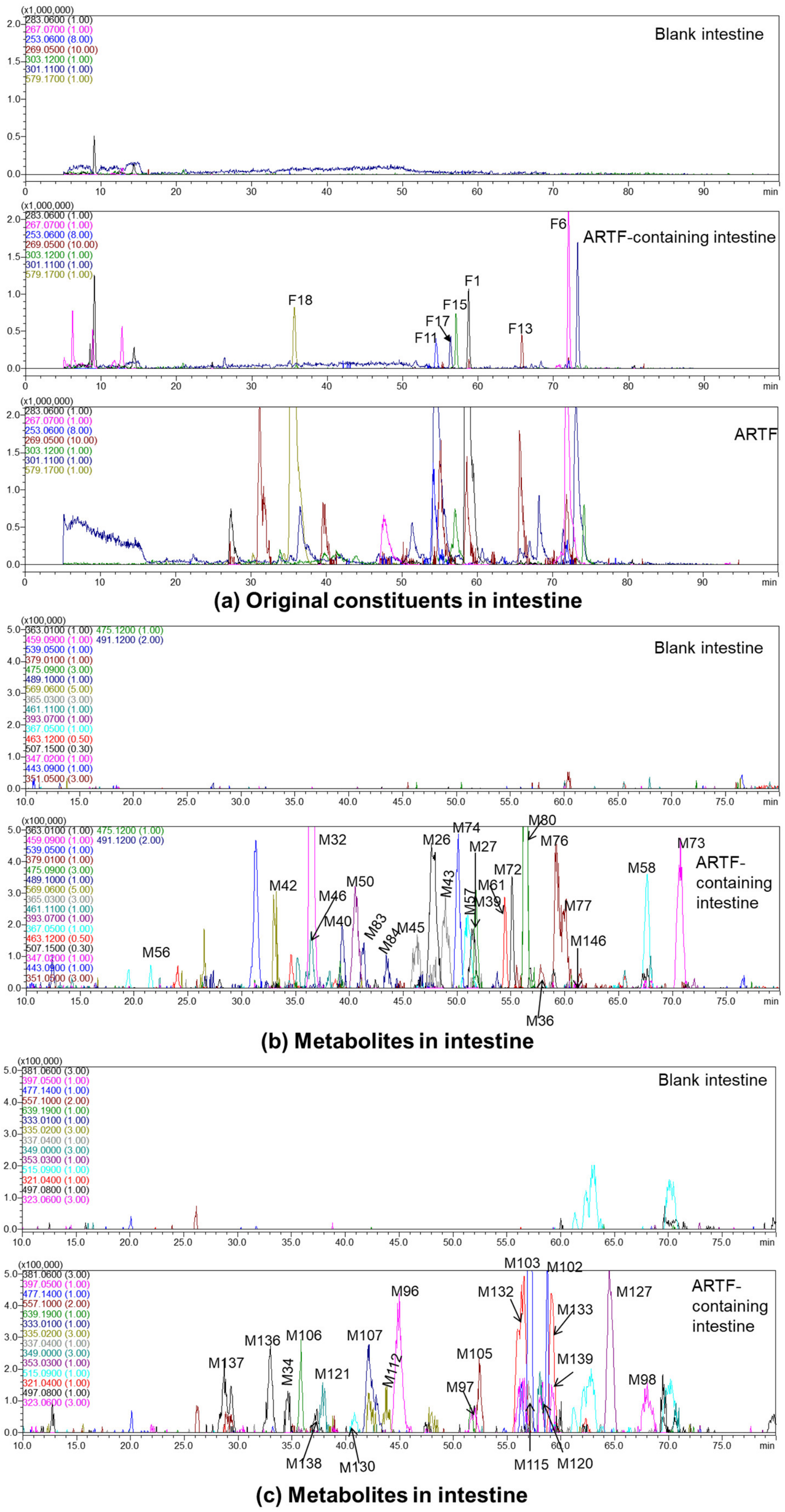

Figure S23. The EICs of original constituents and metabolites distributed in small intestine tissue of rat after administration of ARTF. (a: original constituents; b, c: metabolites).

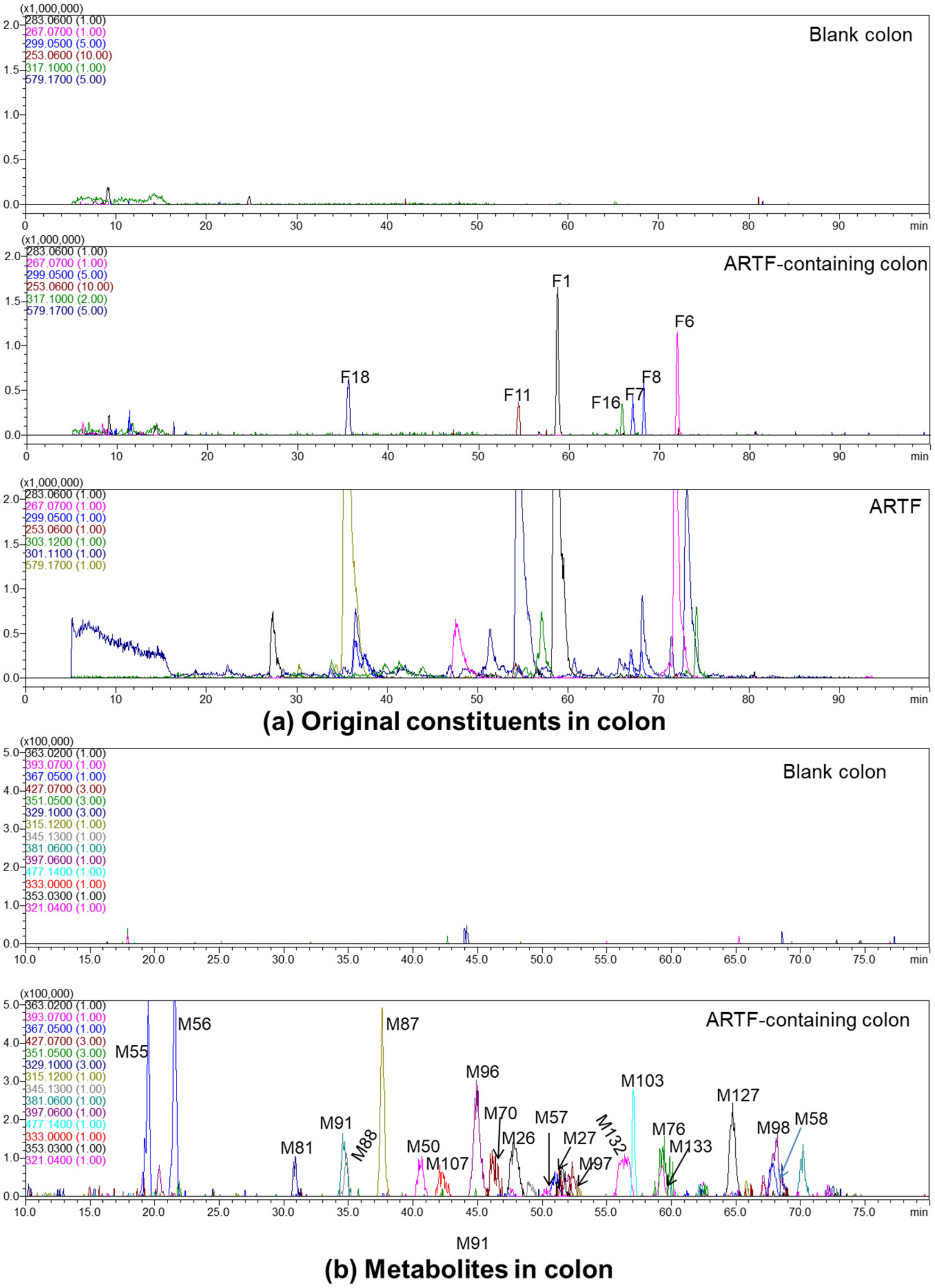

Figure S24. The EICs of original constituents and metabolites distributed in colon tissue of rat after administration of ARTF. (a: original constituents; b: metabolites).

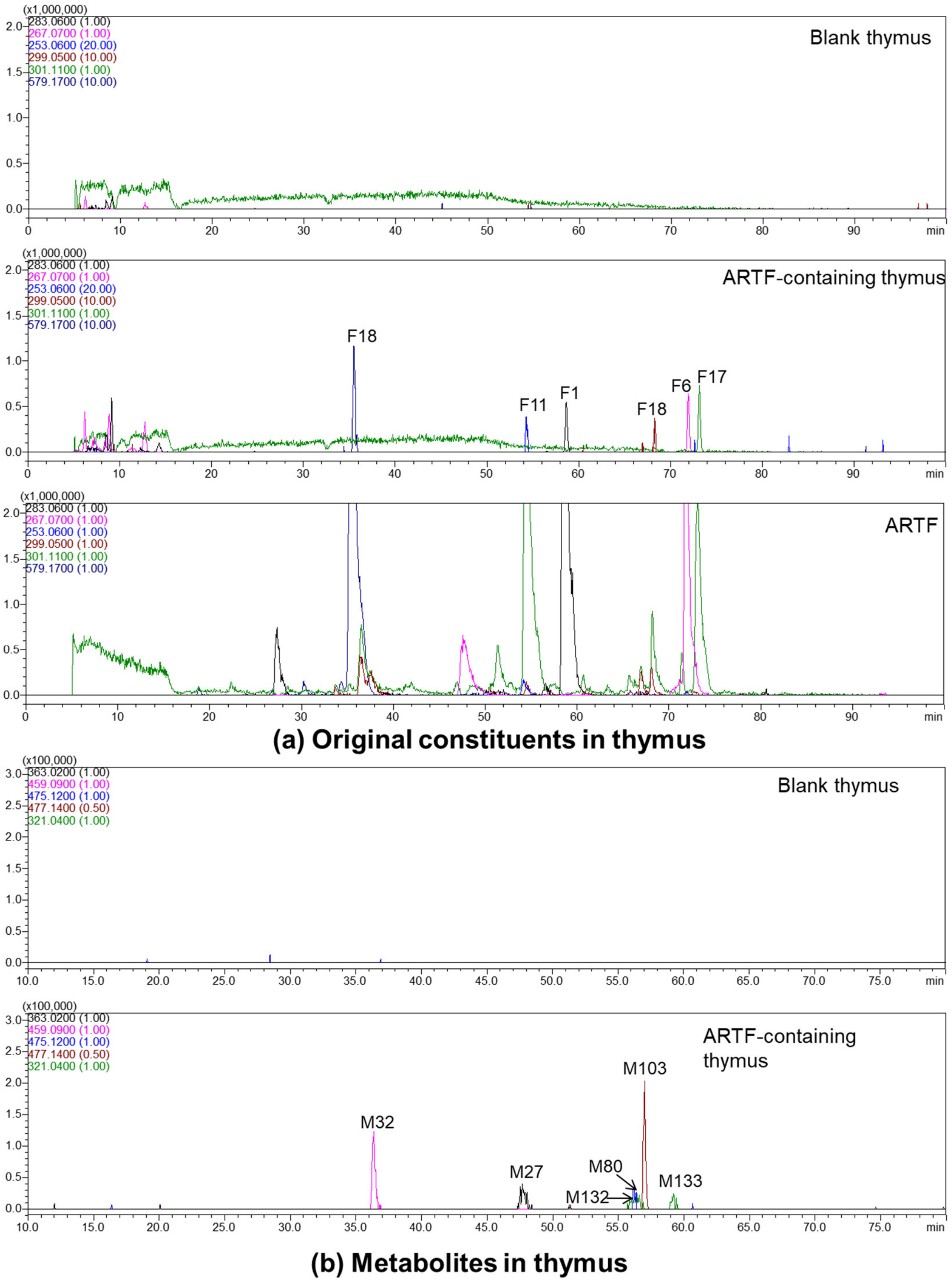

Figure S25. The EICs of original constituents and metabolites distributed in thymus tissue of rat after administration of ARTF. (a: original constituents; b: metabolites).

2.6. NMR Spectroscopy of Isolated Compounds (Figure S26–S29)

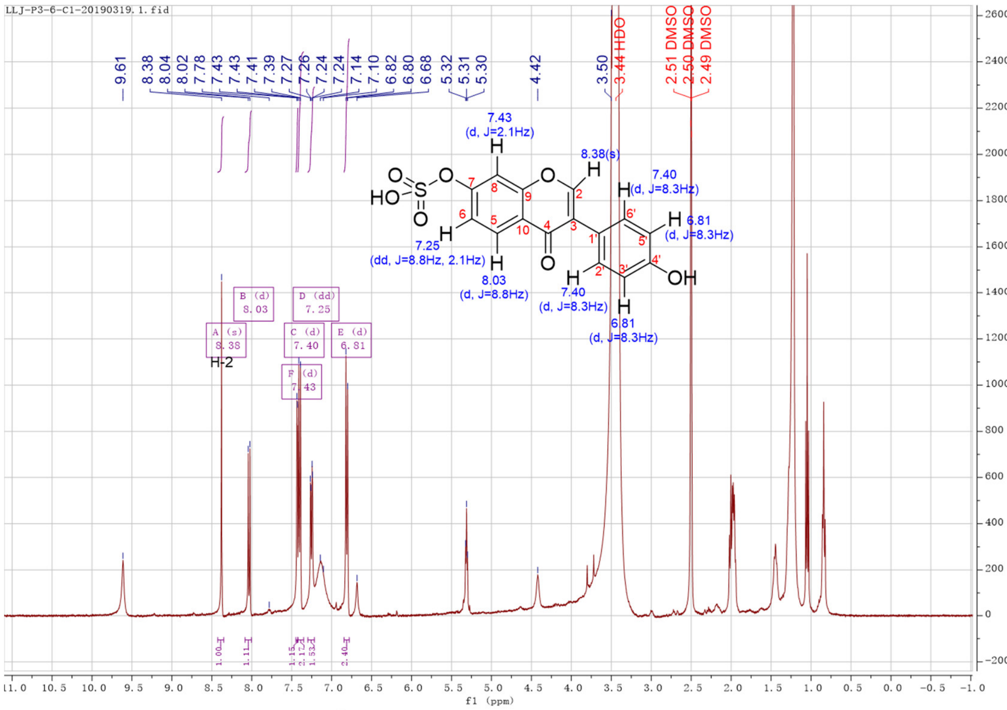

a. ^{1}H-NMR of MI-1 (daidzein-7-O-sulfate)

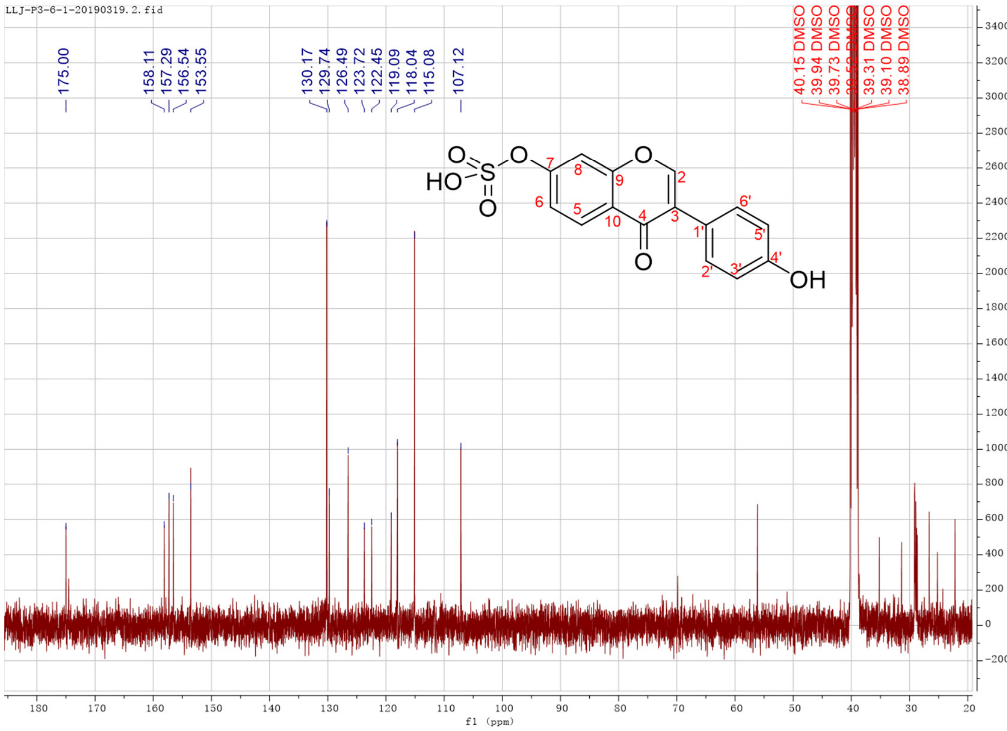

b. ^{13}C-NMR of MI-1 (daidzein-7-O-sulfate)

Figure S26. The NMR spectroscopy of **MI-1 (M108).** (daidzein-7-*O*-sulfate). (a: ^{1}H-NMR; b: ^{13}C-NMR)

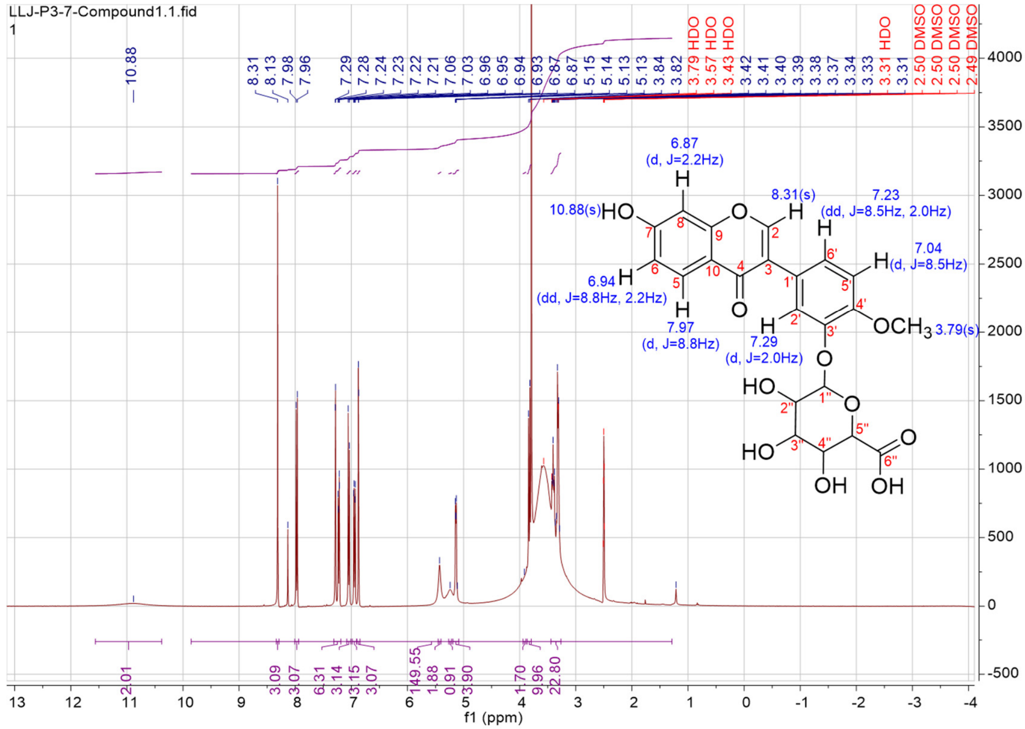

a. ^{1}H-NMR of MI-2 (calycosin-3′-*O*-glucuronide)

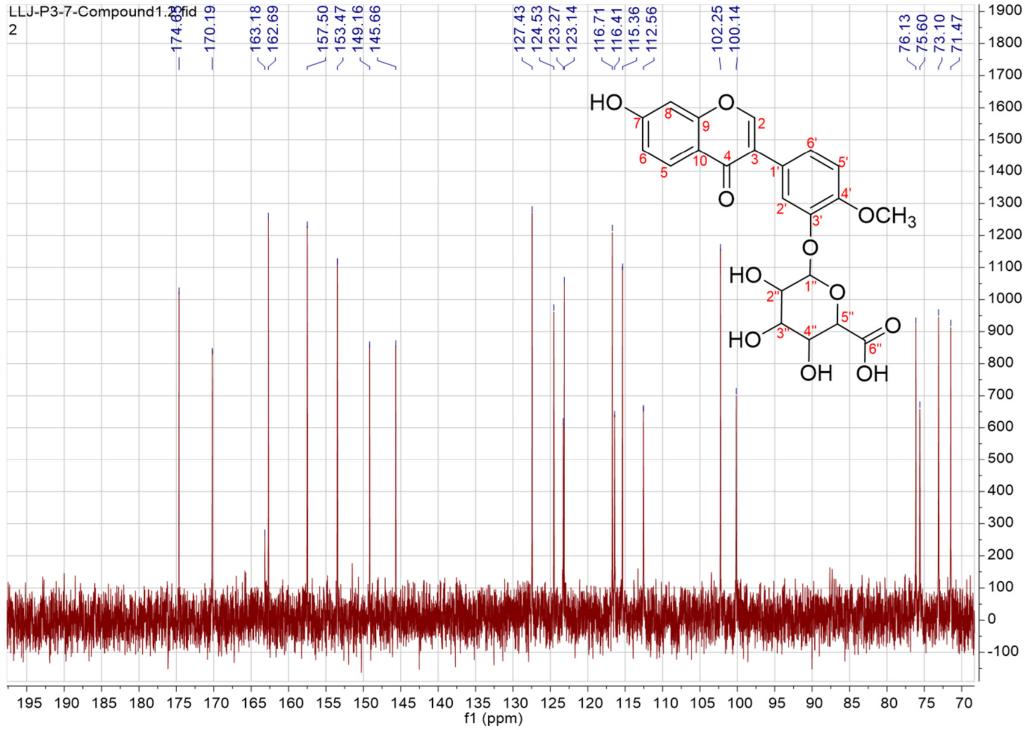

b. ^{13}C-NMR of MI-2 (calycosin-3′-*O*-glucuronide)

Figure S27. The NMR spectroscopy of **MI-2 (M32).** (calycosin-3′-*O*-glucuronide).

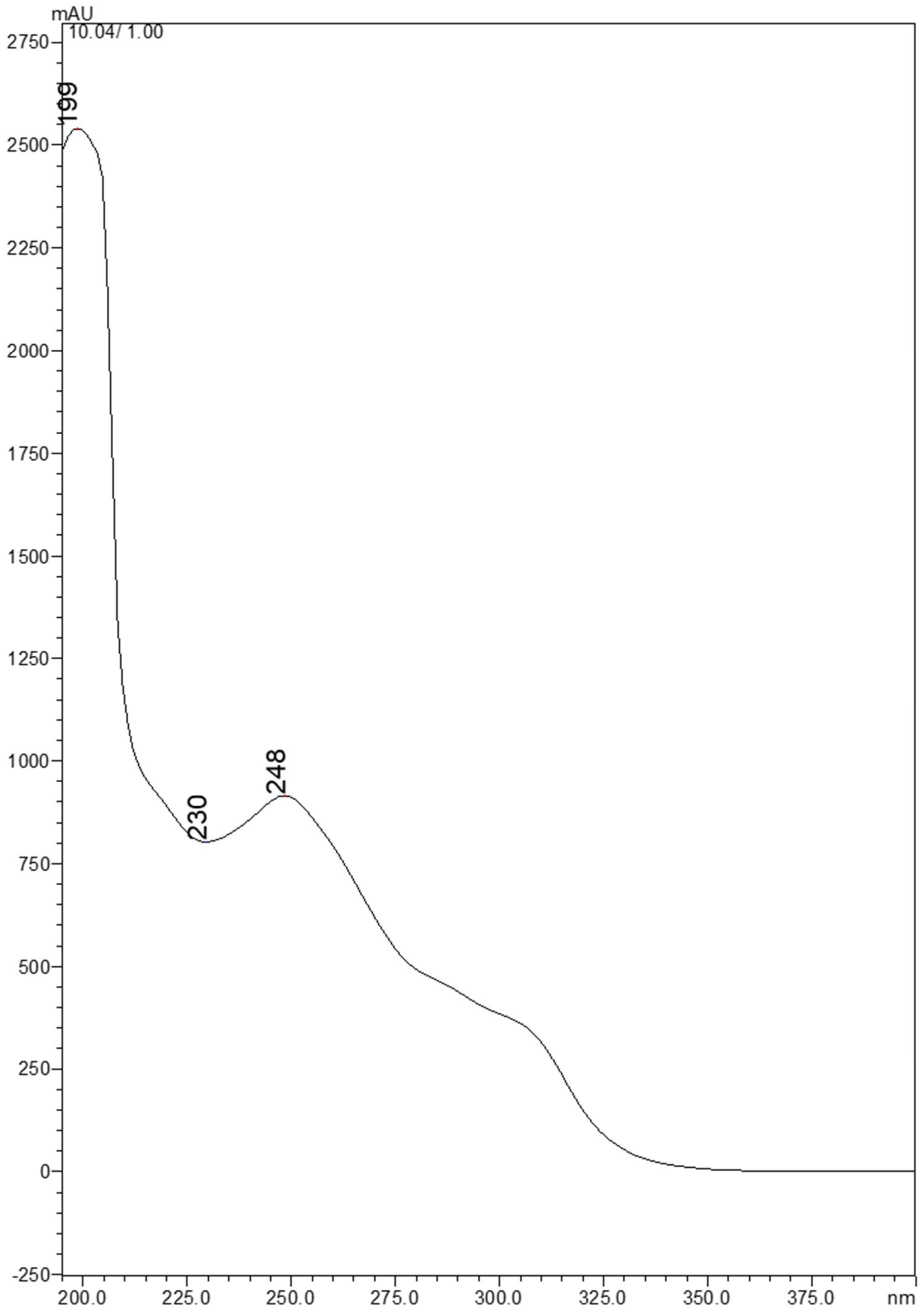

a. UV of MI-3 (calycosin-3′-*O*-glucuronide methyl ester)

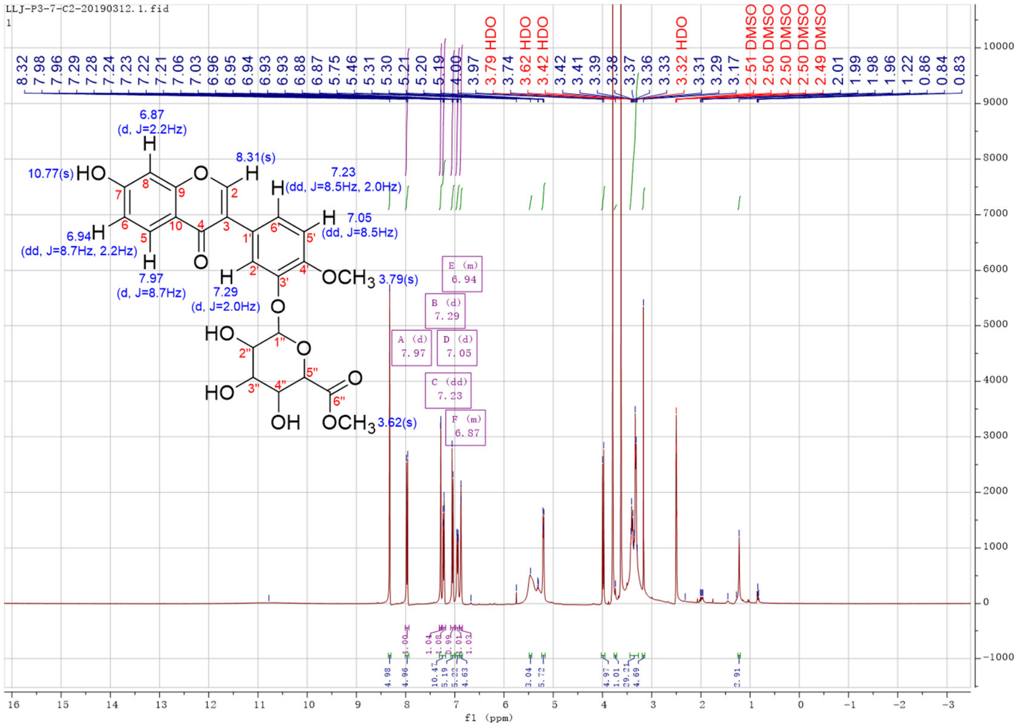

b. ^{1}H-NMR of MI-3 (calycosin-3′-*O*-glucuronide methyl ester)

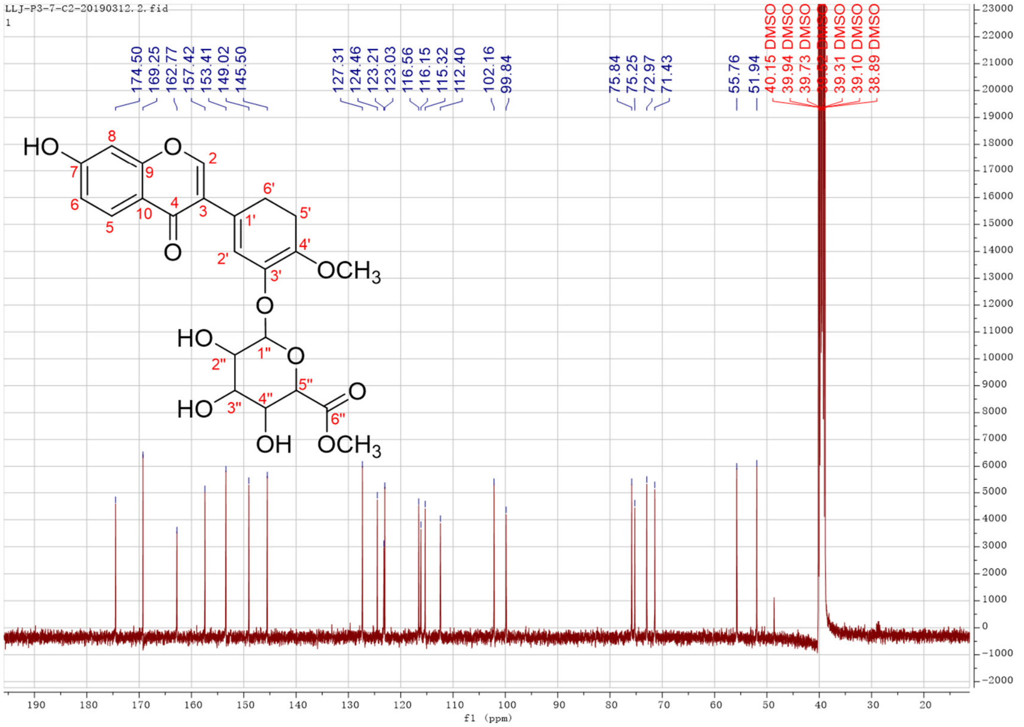

c. ^{13}C-NMR of MI-3 (calycosin-3′-*O*-glucuronide methyl ester)

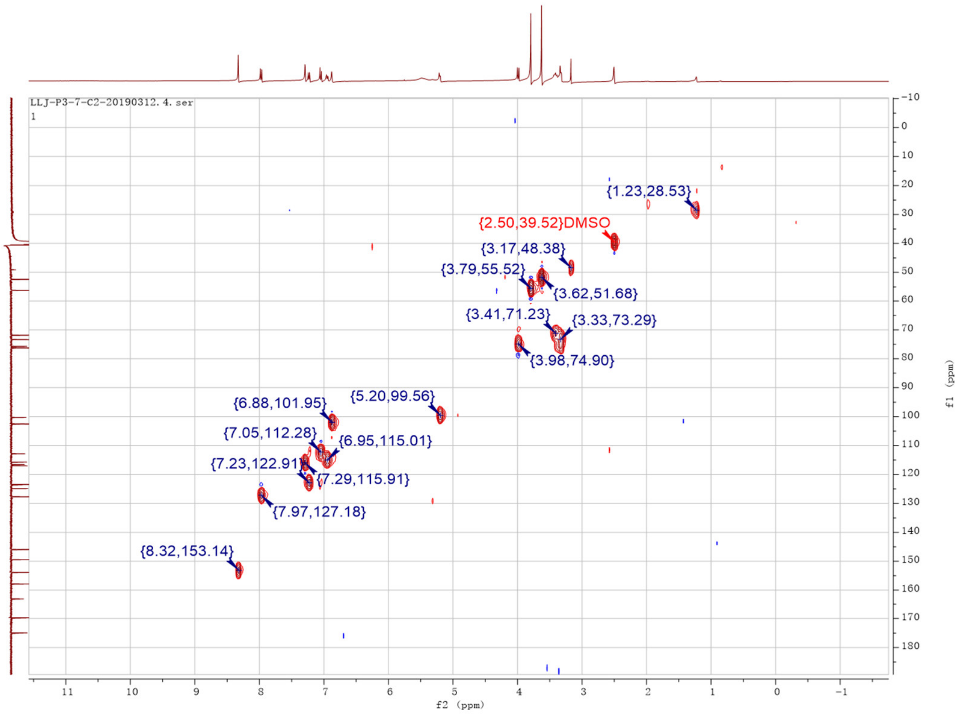

d. HSQC of MI-3 (calycosin-3′-*O*-glucuronide methyl ester)

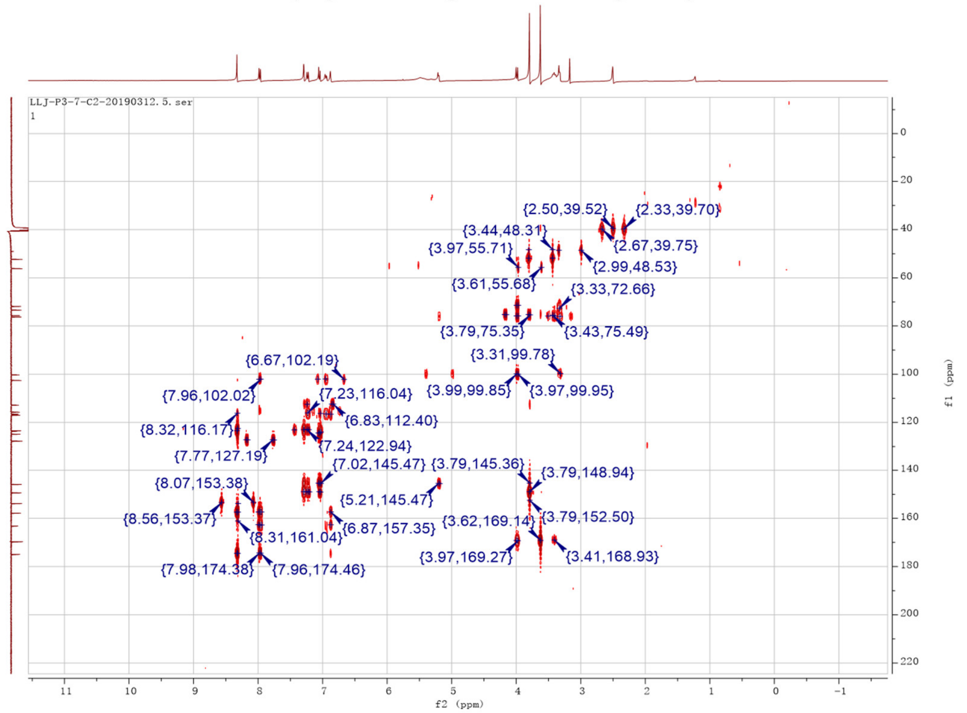

e. HMBC of MI-3 (calycosin-3′-*O*-glucuronide methyl ester)

Figure S28. The NMR spectroscopy of **MI-3.** (calycosin-3′-*O*-glucuronide methyl ester). (a: UV; b: ^{1}H-NMR; c: ^{13}C-NMR; d: HSQC; e: HMBC,).

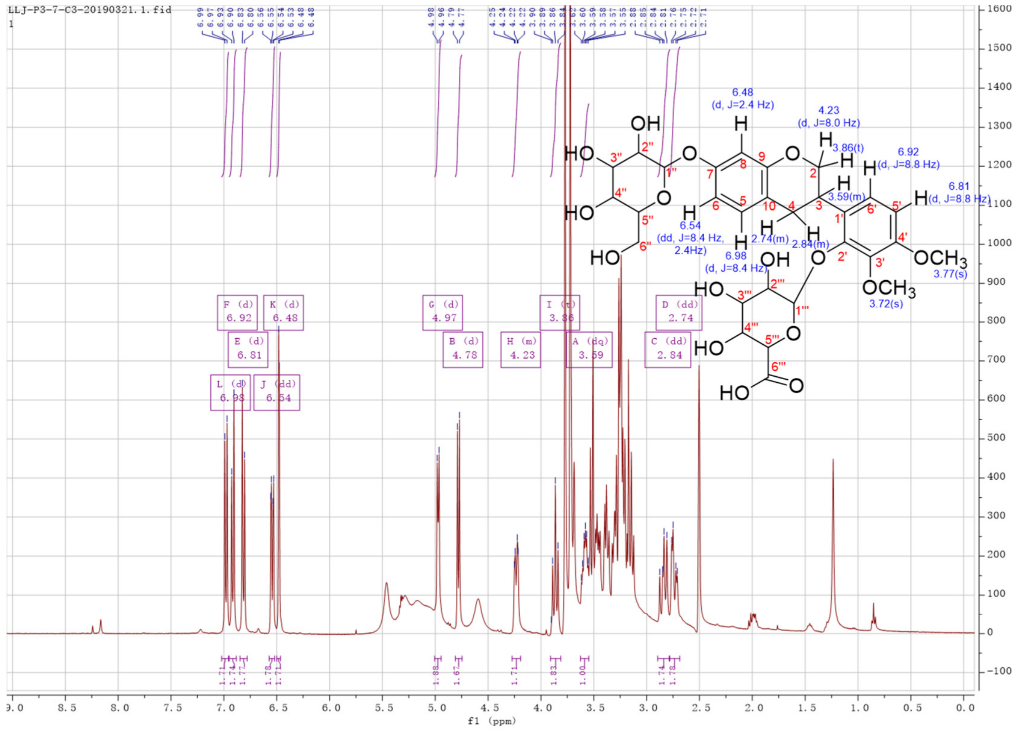

a. ¹H-NMR of MI-4 (astraisoflavan-7-O-glucoside-2′-*O*-glucuronide)

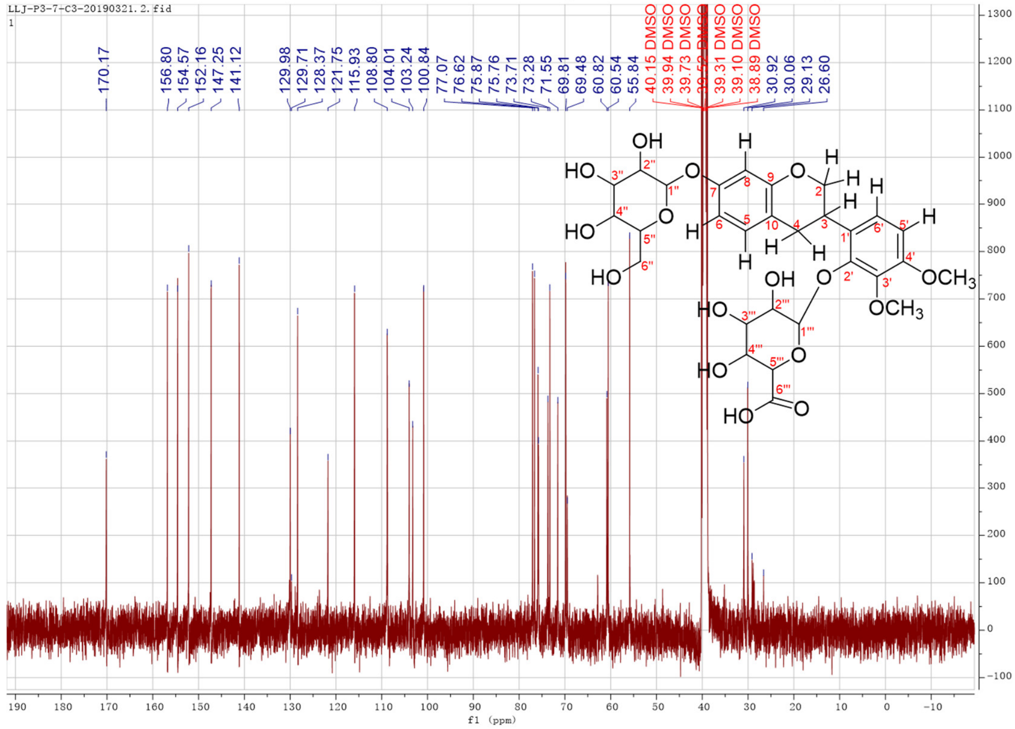

b. ¹³C-NMR of MI-4 (astraisoflavan-7-O-glucoside-2′-*O*-glucuronide)

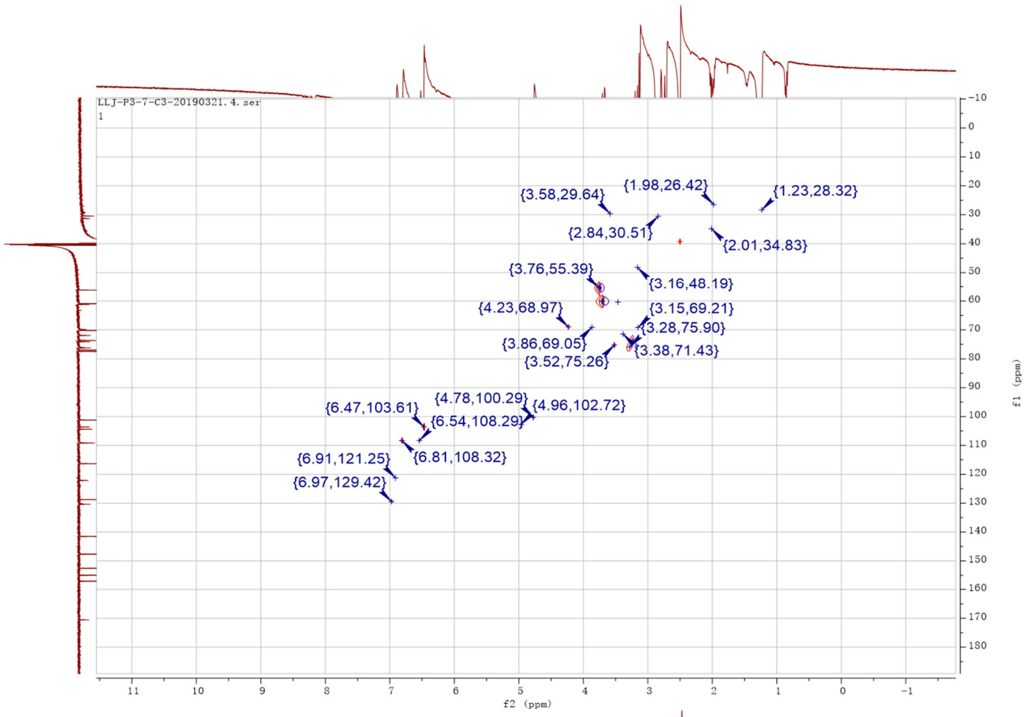

c. HSQC of MI-4 (astraisoflavan-7-O-glucoside-2′-*O*-glucuronide)

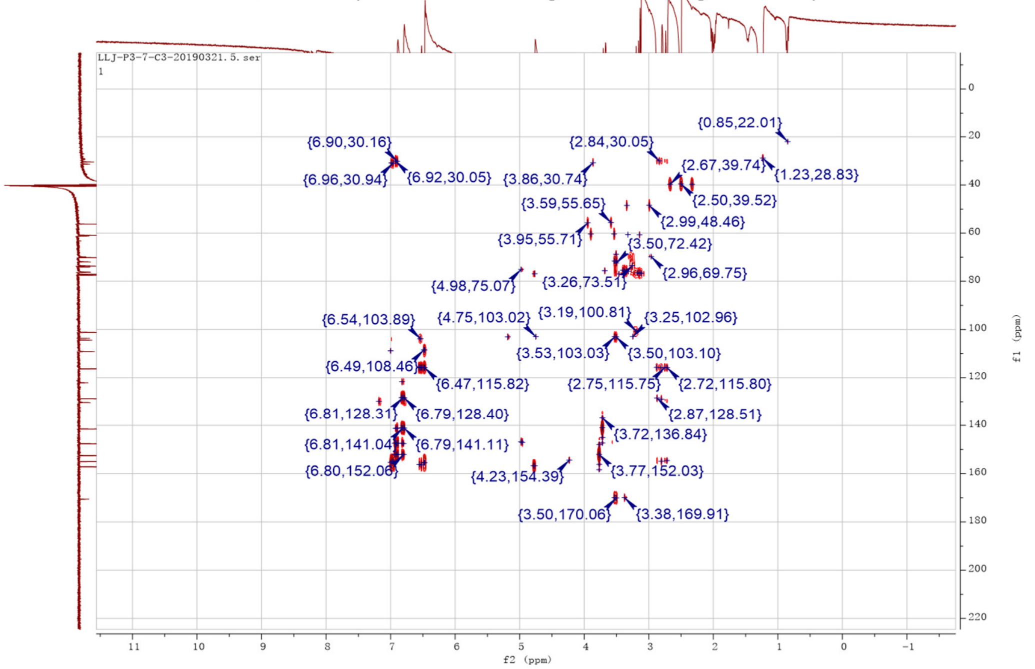

d. HMBC of MI-4 (astraisoflavan-7-O-glucoside-2′-*O*-glucuronide)

Figure S29. The NMR spectroscopy of MI-4. (astraisoflavan-7-*O*-glucoside-2′-*O*-glucuronide). (a: ^{1}H-NMR; b: ^{13}C-NMR; c: HSQC; d: HMBC).

2.7. Chromatogram of ARTF (Figure S30)

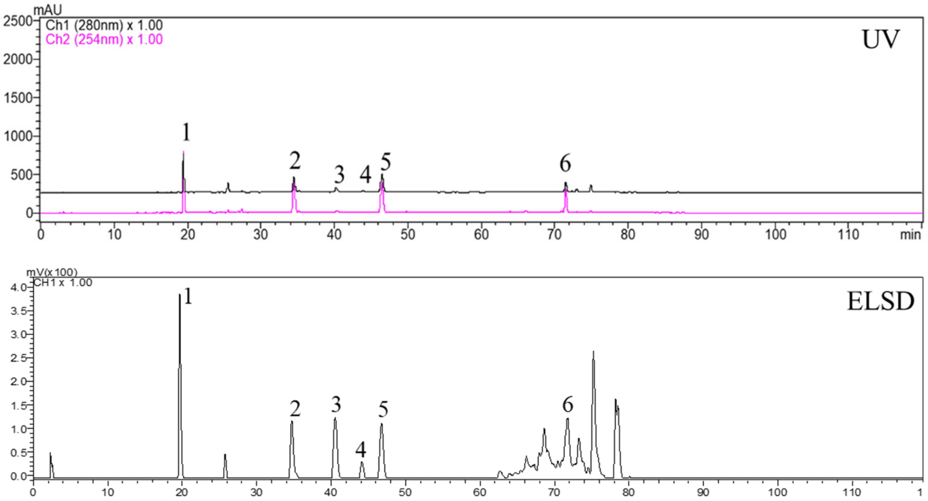

Figure S30. The HPLC-UV-ELSD Chromatogram of ARTF. (1: Calycosin-7-*O*-glucoside; 2: Ononin; 3: Astrapterocarpan-3-*O*-glucoside; 4: Astraisoflavan-7-*O*-glucoside; 5: Calycosin, 6: formononetin).

2.8. Chromatograms of Fraction 1 to Fraction 4 Compared with Blank Urine

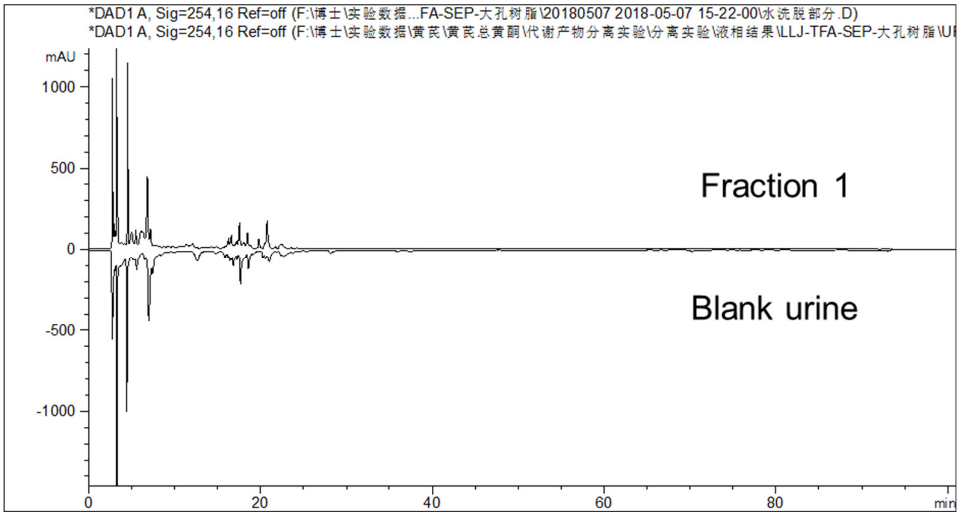

Figure S31. HPLC chromatograms of Fraction 1 and Blank urine.

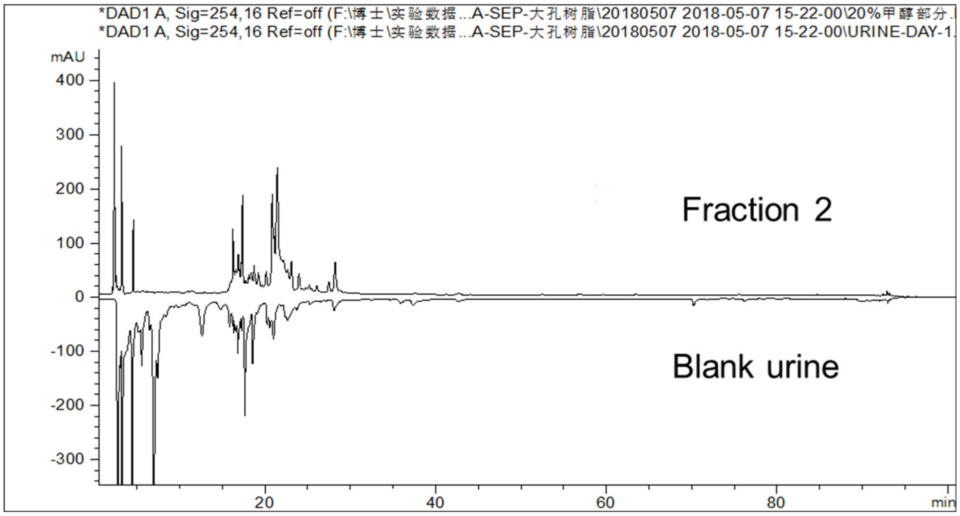

Figure S32. HPLC chromatograms of Fraction 2 and Blank urine.

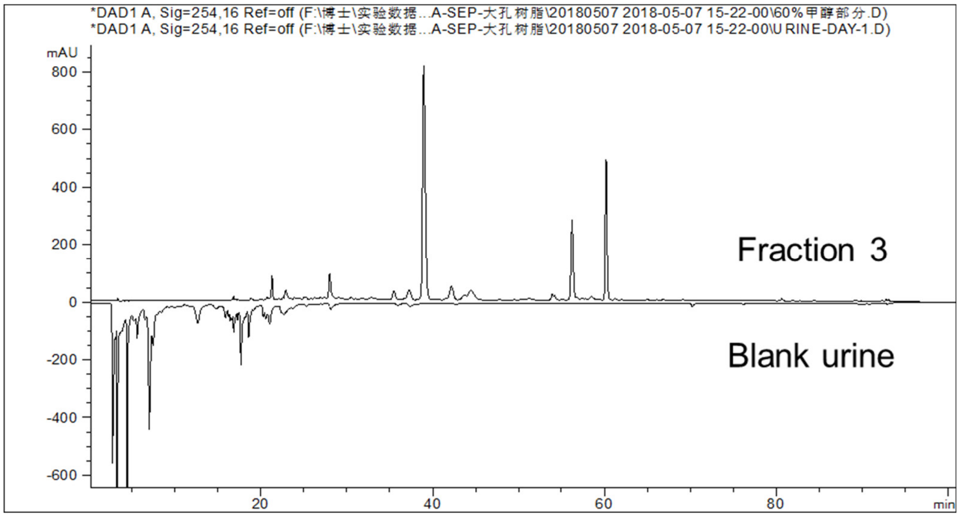

Figure S33. HPLC chromatograms of Fraction 3 and Blank urine.

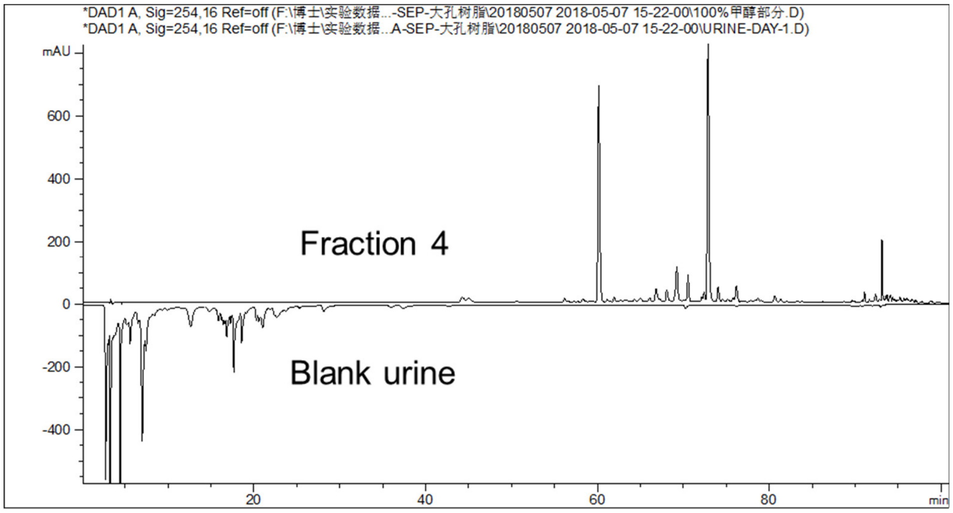

Figure S34. HPLC chromatograms of Fraction 4 and Blank urine..

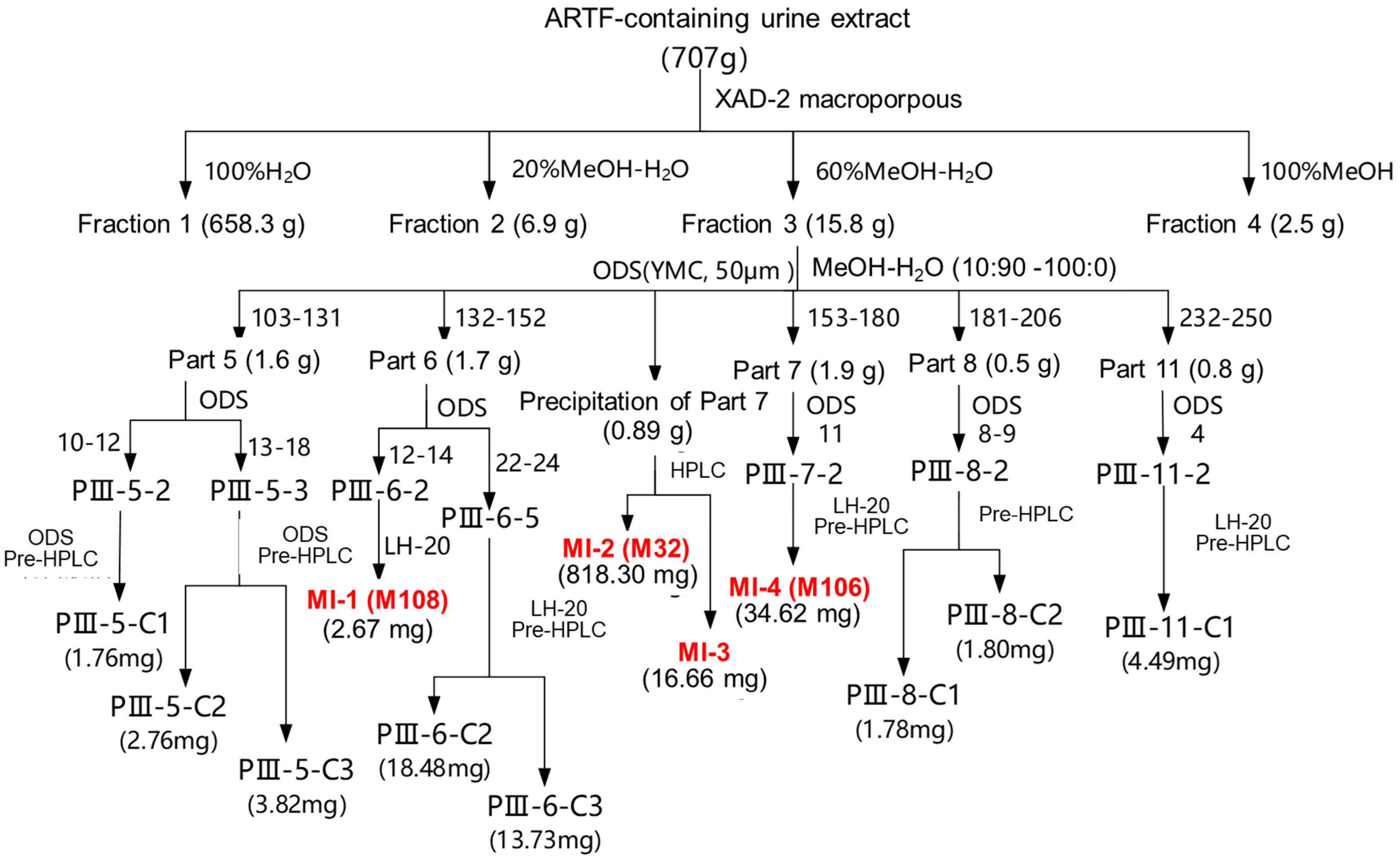

Figure S35. Isolation procedure of ARTF-containing urine.

References

39. Bai, F.; Makino, T.; Kono, K.; Nagatsu, A.; Ono, T.; Mizukami, H., Calycosin and formononetin from astragalus root enhance dimethylarginine dimethylaminohydrolase 2 and nitric oxide synthase expressions in Madin Darby Canine Kidney II cells. *J. Nat. Med.* **2013**, *67*, 782–789.
40. Yu, D.H.; Bao, Y.M.; An, L.J.; Yang, M., Protection of PC12 Cells against Superoxide-induced Damage by Isoflavonoids from Astragalus mongholicus. *Biomed. Environ. Sci.* **2009**, *22*, 50–54.
41. Ma, R.; Yuan, F.; Wang, S.; Liu, Y.; Fan, T.; Wang, F., Calycosin alleviates cerulein-induced acute pancreatitis by inhibiting the inflammatory response and oxidative stress via the p38 MAPK and NF-kappaB signal pathways in mice. *Biomed. Pharmacother.* **2018**, *105*, 599–605.
42. Tian, J.; Wang, Y.; Zhang, X.; Ren, Q.Y.; Li, R.; Huang, Y.; Lu, H.L.; Chen, J., Calycosin inhibits the in vitro and in vivo growth of breast cancer cells through WDR7-7-GPR30 Signaling. *J. Exp. Clin. Canc. Res.* **2017**, *36*, 153–166.
43. Duan, X.; Meng, Q.; Wang, C.; Liu, Z.; Sun, H.; Huo, X.; Sun, P.; Ma, X.; Peng, J.; Liu, K., Effects of calycosin against high-fat diet-induced nonalcoholic fatty liver disease in mice. *J. Gastroenterol Hepatol* **2018**, *33*, 533–542.
44. Ren, M.; Wang, X.; Du, G.; Tian, J.; Liu, Y., Calycosin-7-O-β-D-glucoside attenuates ischemia-reperfusion injury in vivo via activation of the PI3K/Akt pathway. *Mol. Med. Rep.* **2015**, *13*, 633–640.
45. Fu, S.P.; Gu, Y.; Jiang, J.Q.; Chen, X.; Xu, M.J.; Chen, X.M.; Shen, J.G., Calycosin-7-O-beta-D-glucoside regulates nitric oxide/caveolin-1/matrix metalloproteinases pathway and protects blood-brain barrier integrity in experimental cerebral ischemia-reperfusion injury. *J. Ethnopharmacol.* **2014**, *155*, 692–701.
46. Choi, S.I.; Heo, T.R.; Min, B.H.; Cui, J.H.; Choi, B.H.; Park, S.R., Alleviation of osteoarthritis by calycosin-7-O-β-D-glucopyranoside (CG) isolated from Astragali radix (AR) in rabbit osteoarthritis (OA) model. *Osteoarthritis Cartilage* **2007**, *15*, 1086–1092.
47. Zhang, D.M., Effect of calycosin-7-O-beta-D-glucoside on cell apoptosis in cervical cancer HeLa cell and expression of Bcl-2/Bax. *Chin. Tradit. Herbal Drugs* **2015**, *46*, 1498–1502.
48. Zhu, J.T.T.; Choi, R.C.Y.; Chu, G.K.Y.; Cheung, A.W.H.; Gao, Q.T.; Li, J.; Jiang, Z.Y.; Dong, T.T.X.; Tsim, K.W.K., Flavonoids possess neuroprotective effects on cultured pheochromocytoma PC12 cells: A comparison of different flavonoids in activating estrogenic effect and in preventing β-amyloid-induced cell death. *J. Agr Food Chem.* **2007**, *55*, 2438–2445.
49. Dong, L.; Yin, L.; Zhang, Y.; Fu, X.; Lu, J., Anti-inflammatory effects of ononin on lipopolysaccharide-stimulated RAW 264.7 cells. *Mol. Immunol.* **2017**, *83*, 46–51.
50. Jun, M.; Hong, J.; Jeong, W.S.; Ho, C.T., Suppression of arachidonic acid metabolism and nitric oxide formation by kudzu isoflavones in murine macrophages. *Mol. Nutr. Food Res.* **2005**, *49*, 1154–1159.
51. Jin, Y.M.; Xu, T.M.; Zhao, Y.H.; Wang, Y.C.; Cui, M.H., In vitro and in vivo anti-cancer activity of formononetin on human cervical cancer cell line HeLa. *Tumour Biol.* **2014**, *35*, 2279–2284.
52. Yang, S.; Wei, L.; Xia, R.; Liu, L.; Chen, Y.; Zhang, W.; Li, Q.; Feng, K.; Yu, M.; Zhang, W.; Qu, J.; Xu, S.; Mao, J.; Fan, G.; Ma, C., Formononetin ameliorates cholestasis by regulating hepatic SIRT1 and PPARalpha. *Biochem. Biophys. Res. Commun.* **2019**, *512*, 770–778.
53. Applova, L.; Karlickova, J.; Riha, M.; Filipsky, T.; Macakova, K.; Spilkova, J.; Mladenka, P., The isoflavonoid tectorigenin has better antiplatelet potential than acetylsalicylic acid. *Phytomedicine* **2017**, *35*, 11–17.
54. Valeri, A.; Fiorenzani, P.; Rossi, R.; Aloisi, A.M.; Valoti, M.; Pessina, F., The soy phytoestrogens genistein and daidzein as neuroprotective agents against anoxia-glucopenia and reperfusion damage in rat urinary bladder. *Pharmacol Res.* **2012**, *66*, 309–316.
55. He, Y.; Wu, X.; Cao, Y.; Hou, Y.; Chen, H.; Wu, L.; Lu, L.; Zhu, W.; Gu, Y., Daidzein exerts anti-tumor activity against bladder cancer cells via inhibition of FGFR3 pathway. *Neoplasma* **2016**, *63*, 523–531.
56. Wong, M.C.; Portmann, B.; Sherwood, R.; Niemela, O.; Koivisto, H.; Parkkila, S.; Trick, K.; L'Abbe M, R.; Wilson, J.; Dash, P.R.; Srirajaskanthan, R.; Preedy, V.R.; Wiseman, H., The cytoprotective effect of alpha-tocopherol and daidzein against d-galactosamine-induced oxidative damage in the rat liver. *Metabolism* **2007**, *56*, 865–875.
57. Rajadurai, M.; Prince, P.S., Preventive effect of naringin on isoproterenol-induced cardiotoxicity in Wistar rats: an in vivo and in vitro study. *Toxicology* **2007**, *232*, 216–225.
58. Golechha, M.; Chaudhry, U.; Bhatia, J.; Saluja, D.; Arya, D.S., Naringin protects against kainic acid-induced status epilepticus in rats: evidence for an antioxidant, anti-inflammatory and neuroprotective intervention. *Biol. Pharm. Bull.* **2011**, *34*, 360–365.

59. Li, J.; Dong, Y.; Hao, G.; Wang, B.; Wang, J.; Liang, Y.; Liu, Y.; Zhen, E.; Feng, D.; Liang, G., Naringin suppresses the development of glioblastoma by inhibiting FAK activity. *J. Drug Target.* **2017**, *25*, 41–48.
60. Zhou, C.; Lai, Y.; Huang, P.; Xie, L.; Lin, H.; Zhou, Z.; Mo, C.; Deng, G.; Yan, W.; Gao, Z.; Huang, S.; Chen, Y.; Sun, X.; Lv, Z.; Gao, L., Naringin attenuates alcoholic liver injury by reducing lipid accumulation and oxidative stress. *Life Sci.* **2019**, *216*, 305–312.
61. Guo, D.; Wang, J.; Wang, X.; Luo, H.; Zhang, H.; Cao, D.; Chen, L.; Huang, N., Double directional adjusting estrogenic effect of naringin from Rhizoma drynariae (Gusuibu). *J. Ethnopharmacol.* **2011**, *138*, 451–457.
62. Pugazhendhi, D.; Watson, K.A.; Mills, S.; Botting, N.; Pope, G.S.; Darbre, P.D., Effect of sulphation on the oestrogen agonist activity of the phytoestrogens genistein and daidzein in MCF-7 human breast cancer cells. *J. Endocrinol.* **2008**, *197*, 503–515.
63. Kgomotso, T.; Chiu, F.; Ng, K., Genistein- and daidzein 7-O-beta-D-glucuronic acid retain the ability to inhibit copper-mediated lipid oxidation of low density lipoprotein. *Mol. Nutr. Food Res.* **2008**, *52*, 1457–1466.
64. Morito, K.; Aomori, T.; Hirose, T.; Kinjo, J.; Hasegawa, J.; Ogawa, S.; Inoue, S.; Muramatsu, M.; Masamune, Y., Interaction of phytoestrogens with estrogen receptors α and β (II). *Biol. Pharm. Bull.* **2002**, *25*, 48–52.
65. Farina, H.G.; Pomies, M.; Alonso, D.F.; Gomez, D.E., Antitumor and antiangiogenic activity of soy isoflavone genistein in mouse models of melanoma and breast cancer. *Oncol. Rep.* **2006**, *16*, 885–891.
66. Huang, Q.; Huang, R.; Zhang, S.; Lin, J.; Wei, L.; He, M.; Zhuo, L.; Lin, X., Protective effect of genistein isolated from Hydrocotyle sibthorpioides on hepatic injury and fibrosis induced by chronic alcohol in rats. *Toxicol. Lett.* **2013**, *217*, 102–110.
67. Schrader, C.; Ernst, I.M.; Sinnecker, H.; Soukup, S.T.; Kulling, S.E.; Rimbach, G., Genistein as a potential inducer of the anti-atherogenic enzyme paraoxonase-1: studies in cultured hepatocytes in vitro and in rat liver in vivo. *J. Cell Mol. Med.* **2012**, *16*, 2331–2341.
68. Wan, Y.F.; XU, Y.J.; YUAN, B.; WANG, L.L.; Lin, L.Q.; Xu, H.Y., Synthesis of genistein sulfates and effects of genistein and its sulfates on proliferation of breast cancer cells. *Shizhen Guoyi Guoyao* **2011**, *22*, 284–286.
69. Rimbach, G.; Weinberg, P.D.; de Pascual-Teresa, S.; Alonso, M.G.; Ewins, B.A.; Turner, R.; Minihane, A.M.; Botting, N.; Fairley, B.; Matsugo, S.; Uchida, Y.; Cassidy, A., Sulfation of genistein alters its antioxidant properties and its effect on platelet aggregation and monocyte and endothelial function. *Biochim. Biophys. Acta.* **2004**, *1670*, 229–237.

Publisher's Note: MDPI stays neutral with regard to jurisdictional claims in published maps and institutional affiliations.

© 2020 by the authors. Submitted for possible open access publication under the terms and conditions of the Creative Commons Attribution (CC BY) license (http://creativecommons.org/licenses/by/4.0/).
